# Light-driven progesterone production by InP–(*M. neoaurum*) biohybrid system

**DOI:** 10.1186/s40643-022-00575-7

**Published:** 2022-09-01

**Authors:** Kun Liu, Feng-Qing Wang, Ke Liu, Yunqiu Zhao, Bei Gao, Xinyi Tao, Dongzhi Wei

**Affiliations:** State Key Laboratory of Bioreactor Engineering, Newworld Institute of Biotechnology, East China University of Science and Technology, 130 Meilong Road, Shanghai, 200237 China

**Keywords:** Synthetic biology, Progesterone, P450, Electron transfer, InP nanoparticles, NADPH/NADP^+^

## Abstract

Progesterone is one of the classical hormone drugs used in medicine for maintaining pregnancy. However, its manufacturing process, coupled with organic reagents and poisonous catalysts, causes irreversible environmental pollution. Recent advances in synthetic biology have demonstrated that the microbial biosynthesis of natural products, especially difficult-to-synthesize compounds, from building *blocks* is a promising strategy. Herein, overcoming the heterologous cytochrome P450 enzyme interdependency in *Mycolicibacterium neoaurum* successfully constructed the CYP11A1 running module to realize metabolic conversion from waste phytosterols to progesterone. Subsequently, progesterone yield was improved through strategies involving electron transfer and NADPH regeneration. Mutant CYP11A1 (mCYP11A1) and adrenodoxin reductase (ADR) were connected by a flexible linker (L) to form the chimera mCYP11A1-L-ADR to enhance electron transfer. The chimera mCYP11A1-L-ADR, adrenodoxin (ADX), and ADR-related homolog ARH1 were expressed in *M. neoaurum*, showed positive activity and produced 45 mg/L progesterone. This electron transfer strategy increased progesterone production by 3.95-fold compared with *M. neoaurum* expressing mCYP11A1, ADR, and ADX. Significantly, a novel inorganic–biological hybrid system was assembled by combining engineered *M. neoaurum* and InP nanoparticles to regenerate NADPH, which was increased 84-fold from the initial progesterone titer to 235 ± 50 mg/L. In summary, this work highlights the green and sustainable potential of obtaining synthetic progesterone from sterols in *M. neoaurum*.

**Graphical Abstract:**

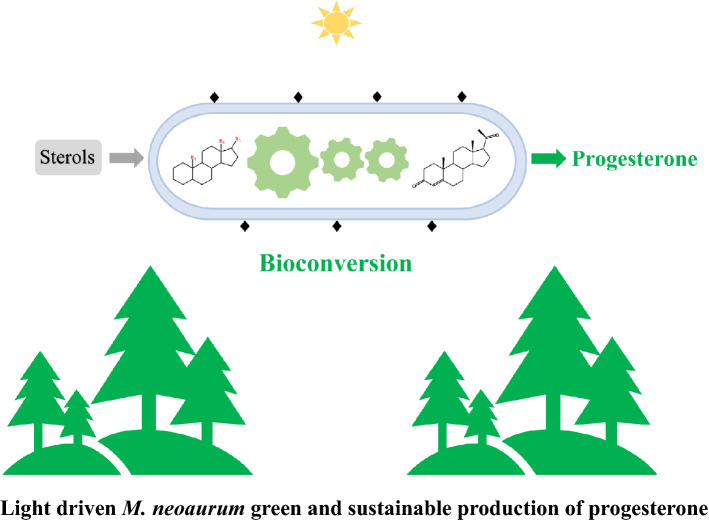

**Supplementary Information:**

The online version contains supplementary material available at 10.1186/s40643-022-00575-7.

## Introduction

Progesterone (4-pregnen-3,20-dione) is a natural progestational hormone necessary for maintaining pregnancy. It not only plays an important role in the reproductive system, such as hormone regulation and sexual response (Mulac-Jericevic et al. 2000), but also affects the respiratory, central nervous, and urinary systems. Traditionally, progesterone is synthesized from the precursor “diosgenin”, which is extracted from plants, such as *Dioscorea zingiberensis*. There are two main routes to synthesize progesterone or other steroid hormones: Route 1 is the “diosgenin to diene” route (Donova and Egorova 2012; Hanson 2005), which involves multistep chemical reactions and microbial transformation processes for the degradation of diosgenin to produce steroid drugs. Another route is “sterol conversion” (Feng et al. 2022; Yao et al. 2014; Zhou et al. 2020), in which diosgenin is used as substrate and transformed into steroid intermediates, and subsequently, by chemical methods, steroid drugs are obtained. These routes inevitably cause environmental pollution, are nongreen, noneconomical, and toxic; waste fresh water resources; and consume unpredictable amounts of organic compounds and heavy metals; thus, a green biotechnological and sustainable concept (Langsdorf et al. 2021) needs to be developed to produce steroid drugs.

Phytosterols (PS) were transformed by *Mycolicibacterium neoaurum* to produce 22-hydroxy-23,24-bisnorchol-4-ene-3-one (HBC), which is a key intermediate in the synthesis of progesterone through two-step chemical reactions: the oxidation of HBC to HBC aldehyde and the copper-mediated catalytic radical oxygenation of aldehyde to progesterone (Peng et al. 2021; Sun et al. 2019). Although this strategy simplifies the synthesis of progesterone from diosgenin, it still uses hazardous chemical reagents, such as 2,2'-bipyridine. Typically, CYP11A1 is the key enzyme that catalyzes cholesterol (ChO) into pregnenolone (Duport et al. 1998), and this reaction, along with 3 O_2_ and 3 NADPH, is carried out in classical steroidogenic glands (Bernhardt and Urlacher 2014). In particular, previous works (Guengerich 2001; Strushkevich et al. 2011) shed light on the catalytic mechanism of CYP11A1, in which the initial hydroxylation reactions at C22 and C20 positions occur, producing 22R-hydroxycholesterol and 20R,22R-dihydroxycholesterol, respectively. The C20-C22 bond of 20R,22R-dihydroxycholesterol was oxidized and cleaved to form pregnenolone with a carbonyl. Based on this mechanism, studies performed over a number of years have demonstrated that a range of substrates can be transformed into novel CYP11A1-derived secosteroids. These analog substrates included 7-dehydrocholesterol (Guryev et al. 2003) and ergosterol (Slominski et al. 2005), which were converted into 7-dehydropregnenolone and pregnenolone, respectively, by purified CYP11A1. CYP11A1 might also efficiently catalyze HBC into progesterone, and progesterone was directly produced from the biodegradation of phytosterols by *M. neoaurum* fermentation. Unfortunately, CYP11A1 has lower activity than desired, mainly because it has heterologous expression interdependency and requires adrenodoxin (ADX) and adrenodoxin reductase (ADR) to receive electrons from NADPH for optimal activity (Grinberg et al. 2000). To overcome CYP11A1 interdependency, Irina A. Pikuleva and coworkers (Pikuleva 2004) identified the putative F-G loop of CYP11A1 associated with membrane attachment. Furthermore, a single amino acid mutation, truncation of the N-terminal, and deletion of the F-G loop together successfully resulted in an approximately fourfold increase in solubility in *E. coli* JM 109 (Janocha et al. 2011). Although these strategies eliminated membrane anchoring to realize free expression in the cytoplasm, CYP11A1 activities were still not high, possibly due to inefficient interaction between CYP11A1 and reductase (Sagadin et al. 2018). Additionally, bovine CYP11A1 and ADX in the reaction system work quite well, but bovine ADR can present a problem because its expression in bacteria is very difficult (Gerber et al. 2015). Therefore, this system should attempt to incorporate other reductases that support the correct reaction and are easy to express in bacteria. Moreover, the efficiency of electron transfer to the overall P450 activities is very often overlooked, and a promising strategy for improving product titers is the optimization of electron flow (Song et al. 2021; Liu and Yu 2020). A protein chimera was applied to the field of a bioenzymatic successfully (Gao et al. 2014). Therefore, a flexible chimera was employed to accelerate electron transfer from NADPH to the substrate in an effort to shorten the distance and increase random collisions or interactions between CYP11A1 and ADR (van Amsterdam et al. 2002).

P450 reactions and/or metabolism involving cofactors, namely, nicotinamide adenine dinucleotide (NADH) or its phosphorylated form (NADPH), present another challenge because the cofactor is expensive and its reactions produce further waste byproducts (Wang et al. 2013). Regardless of the enzymatic or electrochemical regeneration of NAD(P)H, NAD(P)^+^ is reduced to NAD(P)H due to NAD(P)^+^ obtaining a hydride ion [H^−^, (H^+^ + 2e^−^)], whereas the protonation rate of the NAD(P)^+^ radicals is lower than its radical form, leading to inactive 1,6-NAD(P)H and NAD(P)_2_ dimers (Kohlmann et al. 2008; Liu et al. 2018b). These methods would aggravate the metabolic burden for the troublesome P450 and cycle-limiting regeneration of NAD(P)H. Fortunately, inorganic-biological hybrid systems (Brown et al. 2016; Hu et al. 2021; Sakimoto et al. 2015) have established the electron donation capabilities of illuminated semiconductors to regenerate cofactors. Indium phosphide (InP) nanoparticles were selected as a semiconductor photosensitizer in biohybrid systems because they enable the absorption of a greater fraction of the solar spectrum and accept electrons from various microorganisms in the liquid medium (Guo et al. 2018). It is possible that InP–(*M. neoaurum*) biohybrids both intensify the efficiency of NADPH regeneration and enhance the biosynthesis of steroids.

**Fig. 1:**
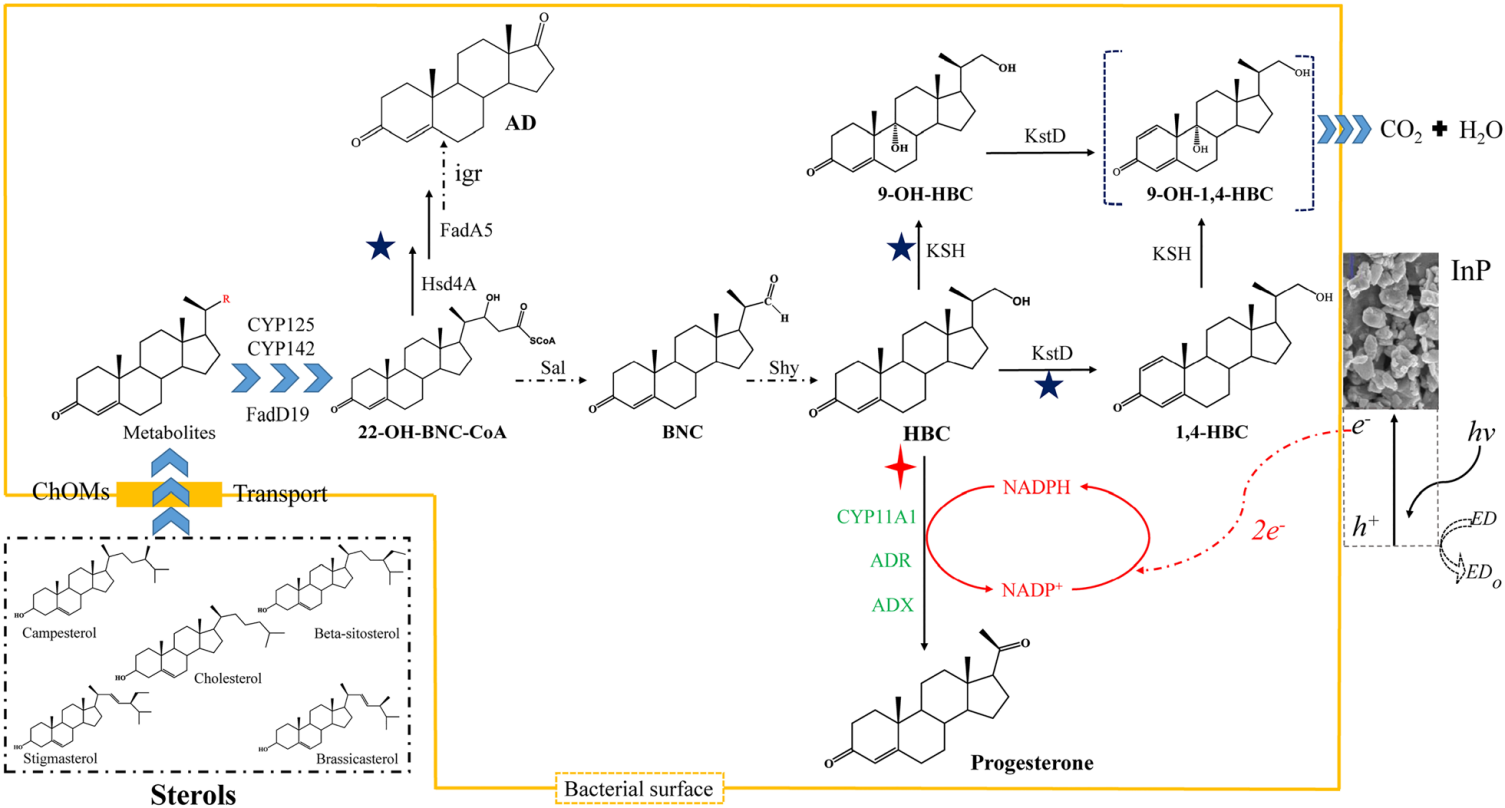
Progesterone biosynthesis schematic in InP–(*M. neoaurum*) biohybrids*.* The schematic depicts an abbreviated overview of the progesterone pathway, beginning with this work’s carbon source sterols. The blue star shows these branch points or building blocks were deleted in this study. The red cross indicates the focus of this study, which is the putative next pathway as performed by the cytochrome P450 (CYP11A1) and its reductase partners. The surface shows the process of cellular NADPH regeneration by photogenerated electrons from InP nanoparticles. h^+^: electron hole, e^−^: electron, ED: electron donors, ED_o_: oxidized electron donors, ChOMs: cholesterol oxidase transport system

In view of the above challenges, sterol conversion, a green and effective strategy, can be directly performed in *M. neoaurum* to obtain progesterone from sterols. Herein, we demonstrate the production of progesterone via *M. neoaurum*, in which the core step is the biotransformation of HBC to progesterone using bovine CYP11A1, thereby enabling the direct production of progesterone from PS (Fig. 1). CYP11A1 expression was optimized by changes including N-terminal truncation, pivotal F-G loop deletion and site mutation (Gerber et al. 2015; Janocha et al. 2011; Pikuleva 2004), and CYP11A1 was successfully expressed in *M. neoaurum* and exhibited positive enzyme activity. Subsequently, to overcome CYP11A1 interdependency with its partners to further improve progesterone titers, the screening of redox partners and construction of enzyme chimeras were used as methods to accelerate electron transfer. Finally, in the context of semiconductor light-harvesting InP nanoparticles, decoupling NADPH regeneration from central carbon metabolism facilitated the production of progesterone. The resulting progesterone production was higher than that reported for *M. smegmatis* mc^2^ 155 cell factories (Strizhov et al. 2014). Overall, this report provides a new *M. neoaurum* for the production of a certain steroid drug, progesterone, from sterols.

## Materials and methods

### Chemicals and reagents

Phytosterols (more than 95%, w/w) (Yao et al. 2013), including campesterol 26.4%, beta-sitosterol 47.5%, stigmasterol 17.7%, and brassicasterol 3.6%, were purchased from Sciphar Natural Products Co., Ltd. (Shanxi, China). Cholesterol, progesterone, tannic acid, poly(allylamine hydrochloride) (PAH) and pregnenolone were supplied by Sigma-Aldrich (Shanghai, China). HBC and InP (3-20 mesh) were obtained from Steraloids (Newport, RI, USA) and Aladdin (Shanghai, China), respectively. HP-β-CD (hydroxypropyl-β-cyclodextrin) was obtained from RSC Chemical Industries Co., Ltd. (Kunshan, China). Other chemicals and reagents were supplied by companies at reagent grade or the highest purity available.

FastDigest restriction enzymes were purchased from Fermentas (Thermo-Fisher, USA). High-fidelity DNA polymerase and NAD(P)H detection kits were obtained from Solarbio Science & Technology Co., Ltd. (Beijing, China). The intracellular concentrations of NADPH and NADP^+^ were determined by a method similar to how NADH was determined by Zhang (Zhang et al. 2009; Zhao et al. 2019). The plasmid extraction kit and gel extraction kit were supplied by Magen Biotech Co., Ltd. (Shanghai, China). A one-step cloning kit was purchased from Yeasen Biotech Co., Ltd. (Shanghai, China).

PS mother liquor (100 g/L) was prepared by mixing phytosterols (20 g), hydroxypropyl-β-cyclodextrin (80 g), and water (100 mL). Then, this turbid liquid was stirred and sonicated to make smaller phytosterols. Finally, the mother liquor was fixed to 200 ml and sterilized at 121 °C for 21 min. ChO and HBC mother liquors (100 g/L) were prepared by the same method.

### Strains, plasmids and primers

For details, please see Additional file 1: Tables S1, S2. *M. neoaurum* ATCC 25795 was isolated from a soil sample using steroids as a single carbon source, which identified sterol consumers with no metabolic pathways in detail. Based on the original strain *M. neoaurum* ATCC 25795, an HBC-producing strain (designated as MNR) was developed by deleting these genes encoding *KstD* (3-ketosteroid-Δ^1^-dehydrogenase), *KSH* (9α-hydroxylase), *Hsd4A* (17β-hydroxysteroid dehydrogenase), and *FadA5* (encoding a thiolase) (Xu et al. 2016).

### Medium and culture conditions

The inoculum was cultured in Luria–Bertani (LB) medium (tryptone 10 g/L, yeast extract 5 g/L, NaCl 10 g/L) or LB plate medium with 1.8% agar. The optimum flask medium (MNR01) was employed as a preliminary experiment in shake flasks, containing 20 g/L glycerol, 2 g/L citric acid monohydrate, 0.5 g/L K_2_HPO_4_·3H_2_O, 0.5 g/L MgSO_4_·7H_2_O, 2.52 g/L KNO_3_, 1.65 g/L (NH_4_)_2_HPO_4_, and 0.05 g/L ammonium ferric citrate with an initial pH of 7.5–8.0. The fermentation medium (MNR02) consisted of 10 g/L glucose, 2.5 g/L citric acid monohydrate, 0.5 g/L K_2_HPO_4_·3H_2_O, 0.5 g/L MgSO_4_·7H_2_O, 3.5 g/L (NH_4_)_2_HPO_4_, and 0.05 ‧/L ammonium ferric citrate and was maintained at pH 7.0 with 2 M NaOH. The MNR01 and MNR02 media were sterilized at 121 ℃ for 21 min and 115 °C for 30 min, respectively.

A loopful of MNR glycerol stock was inoculated into LB plates at 30 °C for approximately 4–6 days. Then, the MNR colonies were picked and cultured in a 20 mL shake flask containing 5 mL of LB medium with 50 μg/mL kanamycin (Kan) for plasmid selection at 30 °C for approximately 2–3 days with shaking at 200 rpm. Primary seed inocula (10%, v/v) were added to a 250 mL shake flask with 30 mL MNR01 medium at 30 °C with shaking at 200 rpm until the optical density was not less than 4 at 600 nm. Finally, the secondary inocula (10%, v/v) were harvested from the 250 mL flask fermentation broth and used to convert sterols. With respect to biohybrid transformation, 20 g/L resting cells converted 5 g/L substrates into progesterone in pH 7.4 phosphate-buffered saline (PBS) buffer under light-emitting diode (LED) illumination in a rotator at 30 °C and 200 rpm.

### Construction of the bacterial operons and strains

All cDNA genes were obtained from NCBI Reference, including bovine CYP11A1 (NP_788817.1), bovine ADR (NP_777116.1), bovine ADX (NP_851354.1), yeast ADR-related homolog (ARH1, AJV00869.1), and porcine ADR (NP_001231656.1). These genes were codon-optimized for *Mycobacterium* and completely synthesized by GenScript (China). In addition, the *g6pdh* gene was amplified from the genome of *M. neoaurum* ATCC 25795. To achieve effective translation in *M. neoaurum*, multiple genes were expressed in tandem by a bacterial operon in the shuttle vector pMV261, and stop codon TGA and RBS sequences were positioned between two adjacent genes. In addition, the linkage of gene fragments in an operon was determined using fusion polymerase chain reaction (PCR). The amplified operons were then ligated to pMV261, which was successively digested by Msc I and Sal I to form the recombinant plasmid pMV261. Additional file 1: Table S1 shows the main plasmids used in this study.

The recombinant plasmid pMV261 with different synthetic bacterial operons was transformed into competent DH5α cells. Then, the recombinant pMV261s were extracted from DH5α cells, and their DNA sequences were sequenced. Finally, these correct recombinant plasmids were electrotransformed into MNRs, and the empty vector pMV261 was also transformed into MNRs as a control. The positive recombinant strains were screened, the presence of their heterologous genes was ensured by PCR, and the strains were kept in 20% glycerol at −80 °C.

### Steroid extraction and analysis

To extract progesterone from the fermentation broth, a 2- to 3-fold volume of ethyl acetate was added to the broth. The broth was dried, oscillated for 3–5 min, and centrifuged at 8000 rpm for 5 min. Finally, the above organic phase was transferred to an empty tube and volatilized in a fume cupboard to remove ethyl acetate, so isopycnic methanol was added to the tube again. Samples and standards were dotted on silica gel plates (Macklin Biochemical Co., Ltd., Shanghai, China), where the developing solvent was an organic mixture of petroleum ether and ethyl acetate (3:2), sprayed with 20% H_2_SO_4_, and subsequently oxidized at 105 °C for 15–20 min for thin layer chromatography (TLC) analysis.

UHPLC–MS (Ultra-Pressure Liquid Chromatography, Mass Spectrometer, Q Exactive Orbitrap, Thermo-Fisher Scientific, USA) analysis was carried out in full scan with electron spray ionization (ESI) by scanning all the ions of the products. The UHPLC separation conditions included ZORBAX SB C18 (2.1 * 150 mm, 3.5 μm, Agilent), acetonitrile (A) and water with 0.1% formic acid (B), 2 μL sample injection, 0.35 mL/min flow rate, and 30 °C column temperature. Gradient elution was performed for 10 min, increasing mobile phase A from 5 to 99%. The MS conditions included ESI, positive and negative ion modes, spray voltage 3500 V (negative ion − 3500 V), capillary temperature 350 °C, sheath gas 40 psi (1 psi ≈ 6.895 kPa) and auxiliary gas 15 psi. A full scan was selected for the ion range from 120 to 1200 m/z. Data were acquired with a Surveyor Autosampler and MS Pump and analyzed with Xcalibur software (Thermo-Fisher Scientific, USA). The product of transformant conversion, progesterone, was confirmed with sample MS analysis and standard (progesterone) MS comparison. The ethyl acetate extract was separated and purified to obtain the collected liquid solution of progesterone by silica gel column chromatography. Then, the organic solvent was removed from the collected liquid by spin evaporation. The residual substance after spin evaporation was dissolved in methanol, and this saturated progesterone solution was placed in a −40 °C refrigerator to crystallize pure progesterone. Finally, ^1^H-NMR and ^13^C-NMR (solution, methanol-*d*_*4*_) spectra were obtained on a Bruker (Ascend 600 MHz) nuclear magnetic resonance (NMR) spectrometer. The progesterone was quantified by high-performance liquid chromatography (HPLC, Agilent 1260). The separation conditions included ZORBAX SB C18 (4.6 * 250 mm, particle size 5 nm, Agilent), CH_3_OH and H_2_O (8:2), 20 μL sample injection, 1 mL/min flow rate, 30 °C column temperature, and an absorbing wavelength of 242 nm.

### InP–(*M. neoaurum*) biohybrid assembly

Indium phosphide nanoparticles were obtained through manual grinding. Field emission scanning electron microscopy (SEM) images were obtained on a GeminiSEM 500 (Germany) to test nanoparticle size, operating at an accelerating voltage of 1–15 kV. The assembly method of InP on cells was based on modified positively charged polymers, poly(allylamine hydrochloride)-containing InP nanoparticles, which were used to adsorb on the *M. neoaurum* surface (Guo et al. 2016). Its morphology was observed by high-resolution transmission electron microscopy (TEM, JEM-2100, Japan). The mixing of the suspension of nanoparticles and cells during the assembly process is described in the literature appendix (Guo et al. 2018).

## Results and discussion

### Overcoming CYP11A1 interdependency: heterologous expression in *M. neoaurum*

**Fig. 2:**
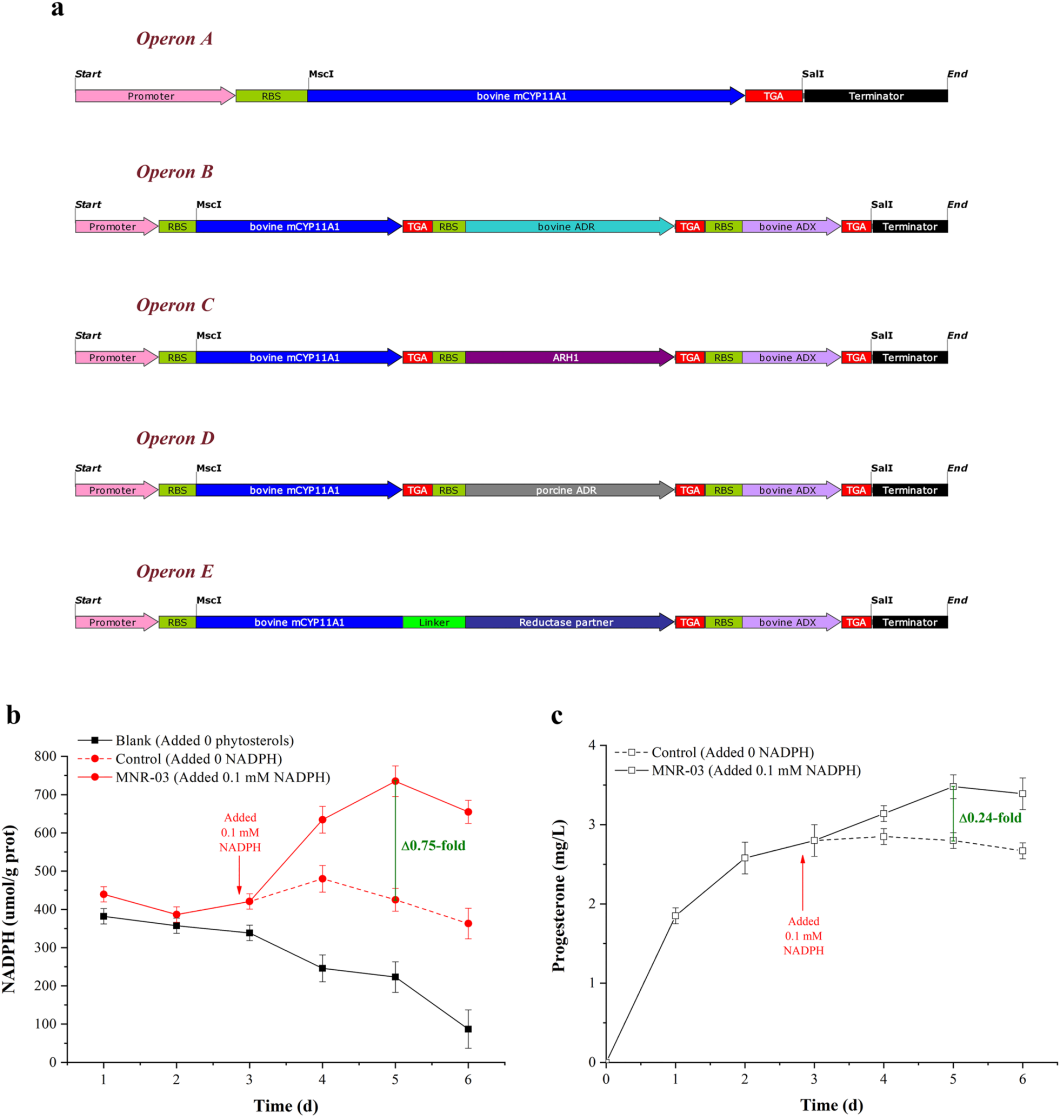
Overcoming the interdependence of P450 catalysis between CYP11A1 and reductase partners in *M. neoaurum*. **a** Overview map of bacterial operon mainly contains genes *cyp11a1* and its partners. RBS: GGAGGAA, Linker (L): (GGGGS)_2_. **b** Intracellular NADPH concentration of MNR-03. **c** Corresponding to progesterone titer of the **b**. The value of the control group was treated as unit one on fermentation of the fifth day. All NADPH assays were performed before bacterial sludge were washed 3 times using PBS buffer and ddH_2_O, respectively

A great deal of P450 heterologous expression involves membrane-bound and specific electron transfer protein(s); in addition, most available methods have difficulty overcoming interdependency. To date, scientists have developed a set of promising devices that combine truncated membrane attachment with directed evolution to obtain free and hydrophilic P450s and successfully increase P450 enzyme activities in multiple hosts (Biggs et al. 2016; Zhang et al. 2019). A simple investigation of free-expressing bovine CYP11A1 was carried out in the MNR, and combined with truncated and site-mutated amino acids, CYP11A1 was found to realize free-expressing CYP11A1 (Additional file 1: Fig. S1). Concretely, the N-terminal truncation, F-G loop deletion, and site mutation K193E of CYP11A1 were modified, but the conserved amino acids of the region of ADX binding to CYP11A1 (465R, 466R) ensured consistency (Additional file 1: Fig. S2). In addition, genes with high GC (65% to 70%) near that of *M. neoaurum* ATCC 25795 (~ 66.7%) were also optimized, and software predicted that its mRNA secondary structure was simpler, with fewer hairpin residues and higher free energy, indicating that mutant *cyp11a1 (mcyp11a1)* translation would be easier. mCYP11A1 was free and soluble, indicating that the production of progesterone achieved the first step in the P450 module for *M. neoaurum* metabolic conversion. The P450 module was added downstream of producing HBC (Fig. 1) and was found to inhibit *M. neoaurum* growth and decrease the HBC titer (unpublished data). The introduction of multiple plasmids pMV261 with different resistance resulted in metabolic burden (Karim et al. 2013), the burdensome plasmid pathway was subjected to unknown inactivation, and the introduction of a multiple plasmid system aggravated this burden, especially for the troublesome metabolism of P450. Additionally, the genome editing tools of *M. neoaurum* are immature so that multiple genes cannot be localized to multiple sites, only the *attB* site. Therefore, to prevent multiplasmid system metabolic burden, the P450 module pathway involving enzymes was tandemly expressed in a bacterial operon (Fig. 2a).

Subsequently, to test CYP11A1 module metabolic pathway running status, the substrates PS and ChO were added to flasks for fermentation, respectively. The metabolite progesterone was initially analyzed and compared by chromatography. ChO was used as a substrate because the conversion rate of its utilization by MNRs is higher than that of PS, making it easier to analyze the target product (Additional file 1: Fig. S3). The Rf and RT corresponding to the dots and peaks of the new product were consistent with the position of standard progesterone (Additional file 1: Fig. S3, S4). More interestingly, HBC was added to the medium and fermented by MNR-03, and merely expressing mCYP11A1 in the MNR was found to convert HBC into progesterone. The cause might be that the chassis has soluble electron carriers from NADPH-dependent reductase to P450, which performs the same function of transmitting electrons as ADR and ADX. To address this discovery theoretically, *M. neoaurum* has diversiform P450s (~ 30) and electron carriers (Liu et al. 2018a). In addition, the natural redox partners of P450s are mostly unknown, and P450s can also accept electrons from other partners (Sagadin et al. 2018). In practice, class I P450 enzymes that catalyze steroids abide by the following reaction (Sadeghi and Gilardi 2013):

$${\text{R-H}} + 3{\text{NAD(P)H}} + 3{\text{O}}_{2} + X{\text{H}}^{ + } \mathop{\longrightarrow}\limits_{{{\text{ADR}}\,{\text{ADX}}}}^{{{\text{CYP11A1}}}}{\text{R-OH + 3NAD(P)}}^{ + } + x{\text{H}}_{2} {\text{O}}$$

The quantitative relationship between the produced progesterone and intracellular consumed NADPH revealed the existence of unknown electron carriers consistent with ADR and ADX functions in MNRs. The results suggested that the ratio of progesterone to NADPH (1:3.125) was close to the theoretical value of 1:3 (Fig. 2b, c). Therefore, this result indicated that abundant P450 enzymes and electron carriers in MNRs support electron transfer for CYP11A1. In total, *M. neoaurum* has the advantage of transporting electrons between NADPH and CYP11A1 and overcomes the interdependence of CYP11A1 with the native host.

### Identification of biotransformation products

**Fig. 3:**
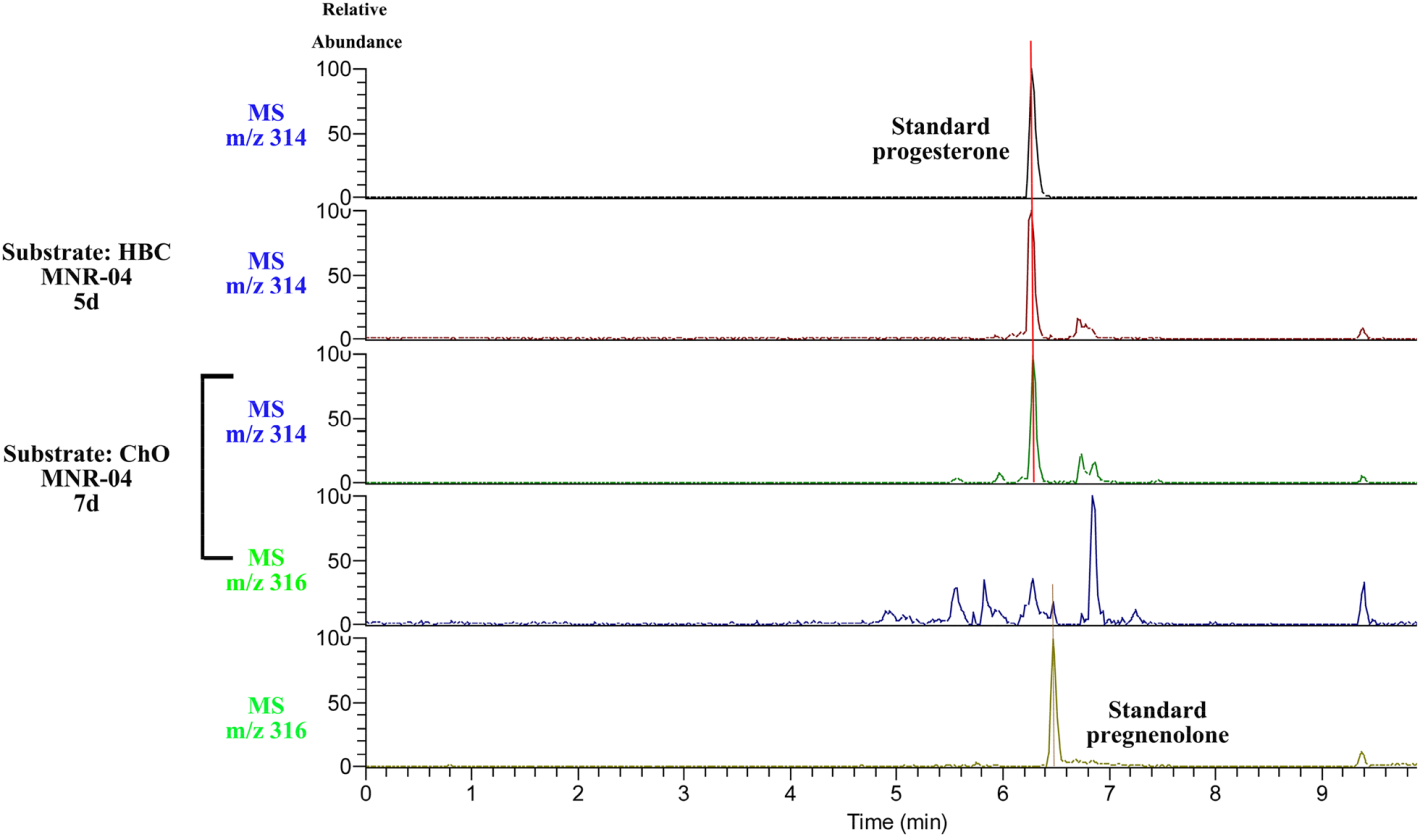
Analysis of products by high-resolution mass spectrometry. The substrates HBC and cholesterol (ChO) were biotransformed by MNR-04 fermentation, respectively

The bioconversion products, using HBC and ChO as substrates, were further characterized by high-resolution mass spectrometry (HRMS). As shown in Fig. 3, the HRMS results revealed that the mass-to-charge ratios of the bioconversion products progesterone and pregnenolone were 314 m/z and 316 m/z, respectively, and these products were equal to preestablished standards (Additional file 1: Figs. S5–S7). To further confirm the structure of the product, the isolated and purified product was subjected to structural analysis by NMR, and ^1^H-NMR and ^13^C-NMR suggested that this product was progesterone (Additional file 1: Figs. S8, S9). In addition, pregnenolone was thought to be the main product of CYP11A1 transforming ChO because reports have confirmed that CYP11A1 converts ChO to pregnenolone (Gerber et al. 2015; Makeeva et al. 2013; Strizhov et al. 2014). Unexpectedly, the HPLC results suggested that progesterone was the main product and that pregnenolone was an intermediate in the conversion of ChO to progesterone. Therefore, HBC is catalyzed by CYP11A1 to produce progesterone, and ChO is directly metabolized into progesterone by MNR-04. With respect to pregnenolone, one hypothesis is that 3β-HSD (3beta-hydroxysteroid dehydrogenase) catalyzes C3 dehydrogenation from pregnenolone, leading to the production of progesterone (Szczebara et al. 2003; Xu et al. 2016). In total, progesterone was directly obtained from PS in engineered *M. neoaurum*.

### Intermolecular electron transfer driven by flexible chimera

**Fig. 4:**
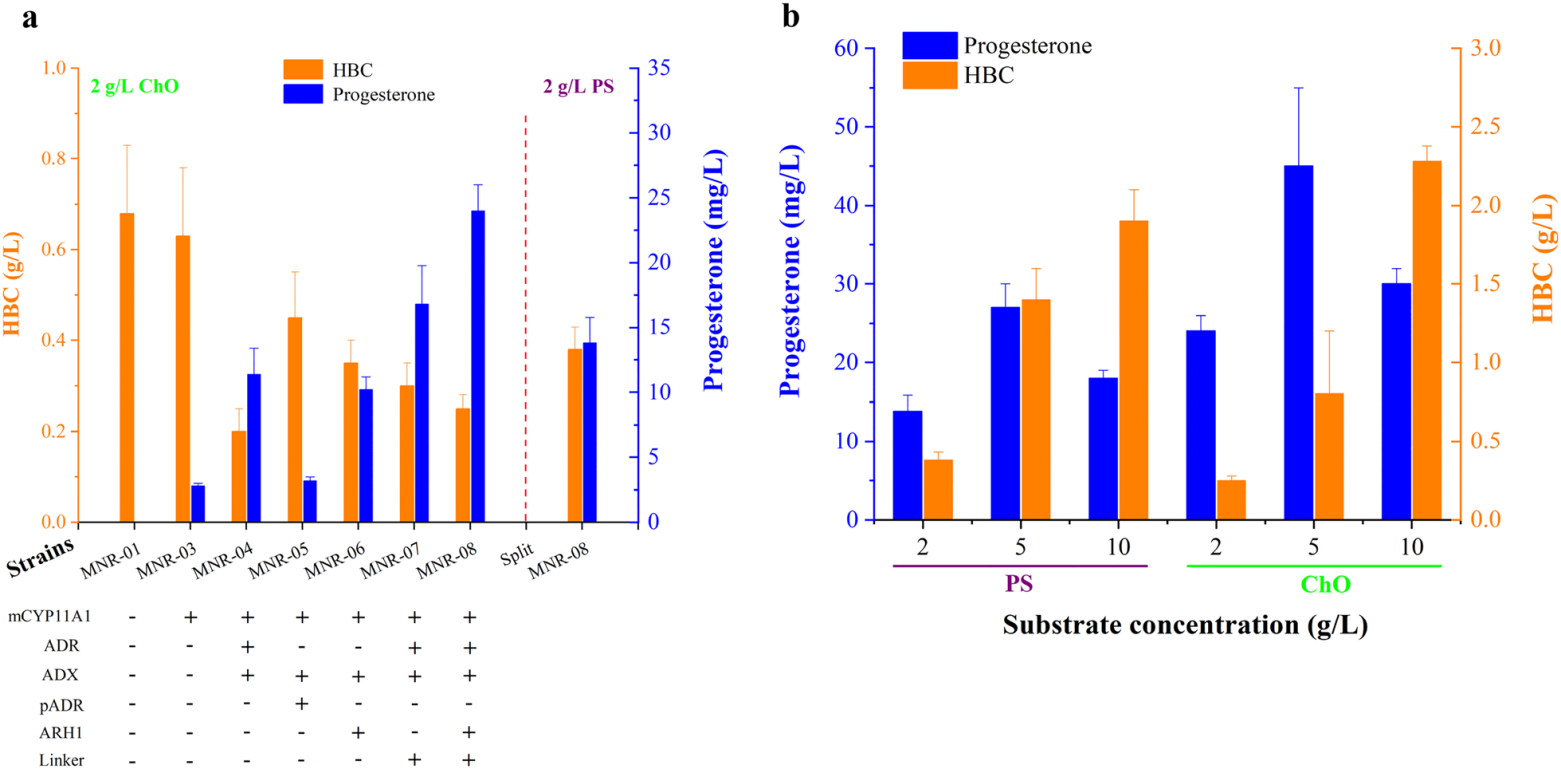
**a** P450 module optimization screen. For strain details, please see appendix, Additional file 1: Table S1. **b** The effect of substrates on progesterone production in pH 7.4 PBS by 20 g/L resting MNR-08 conversion for 5 days

Reports addressing intermolecular electron transfer for P450 have four main classes of approaches to enhance electron transfer, including screening of redox partners, artificial fusion chimera, and light-activation as well as electrochemical reductions. With respect to screening redox partners, it is very difficult to establish high-throughput screening technology. Therefore, known oxidoreductase chaperones were expressed, such as porcine ADR (MNR-05) pADR) and *S. cerevisiae* ARH1 (MNR-06): the former showed negative titers, but the latter showed positive activities (Fig. 4a). This result may be because ARH1 is an ADR-related homolog derived from microbes. Furthermore, the rat CYP1A1-cytochrome P450 reductase (CPR) fusion protein, the first microsomal P450-CPR fusion protein, was successfully constructed by Murakami et al. in (1987), indicating efficient electron transfer between domains within artificial fusion, and this strategy could be a promising application in biotechnology. Subsequently, many reports studied P450 electron transfer using artificial fusion, which included bovine CYP17A1-Rat CPR (Fisher et al. 1992) and CYP3A4-BMR (Dodhia et al. 2008). Without the exception of CYP11A1, Jennifer and coworkers (Harikrishna et al. 1993) constructed three fusion proteins and transfected them into COS-1 cells; only the CYP11A1-ADR-ADX fusion protein produced substantially more pregnenolone. In contrast, the artificial chimera CYP11A1-L-ADR (MNR-07) showed positive activity, 16.8 mg/L progesterone, leading to an over 1.47-fold improvement over MNR-04. The direct fusion of CYP11A1-ADR could impact the interactions of domains; however, the linker [L: (GGGGS)_2_] (Chen et al. 2013) provided flexibility and allowed for mobility of the connecting functional domains, resulting in close and correct domains based on native interactions of CYP11A1 and ADR. The close and correct domains indicated that their electron transfer would yield higher effectivity and activity. ADX exhibits a cytosolic distribution and is easily expressed in bacteria (Gerber et al. 2015), so ADX probably collides and interacts with CYP11A1, causing electron transfer from ADX to CYP11A1. Therefore, the construction of chimera CYP11A1-L-ADR-L-ADX would not significantly improve electron transfer. In addition, electron flow was enhanced by overexpressing ARH1, which can supply electrons for CYP11B1 to enhance steroid production and promote metabolic flow toward hydrocortisone (Szczebara et al. 2003). The electron flow was enhanced through coexpression of ARH1 in MNR-08 so that the progesterone titer reached 24 mg/L. To further improve the progesterone titer, PS and ChO were compared by resting cells of MNR-08 (Zhang et al. 2020; Caro et al. 2007; Wu and Ng 2017), and 45 mg/L progesterone was obtained using 5 g/L ChO as a substrate for fermentation (Fig. 4b), which was 16.1 times higher than the initial concentration. However, the transformation of sterols into progesterone did not abide by mass balance; progesterone increased by approximately 20 mg/L, leading to its precursor HBC decreasing by more than 20 mg/L (progesterone/HBC = 330/314 ≈ 1/1, w/w) (Fig. 4a). This result was caused by 3 product-related aspects, including bacteriostasis, decomposition, and feedback inhibition (Additional file 1: Figs. S10, S11). As we reported previously, inhibition of yeast growth by progesterone caused a sharp drop in fluorescence intensity (Liu et al. 2022). These problems are expected to be solved later by product separation in situ and stress screening.

### Preparation and characterization of the biohybrid system

**Fig. 5:**
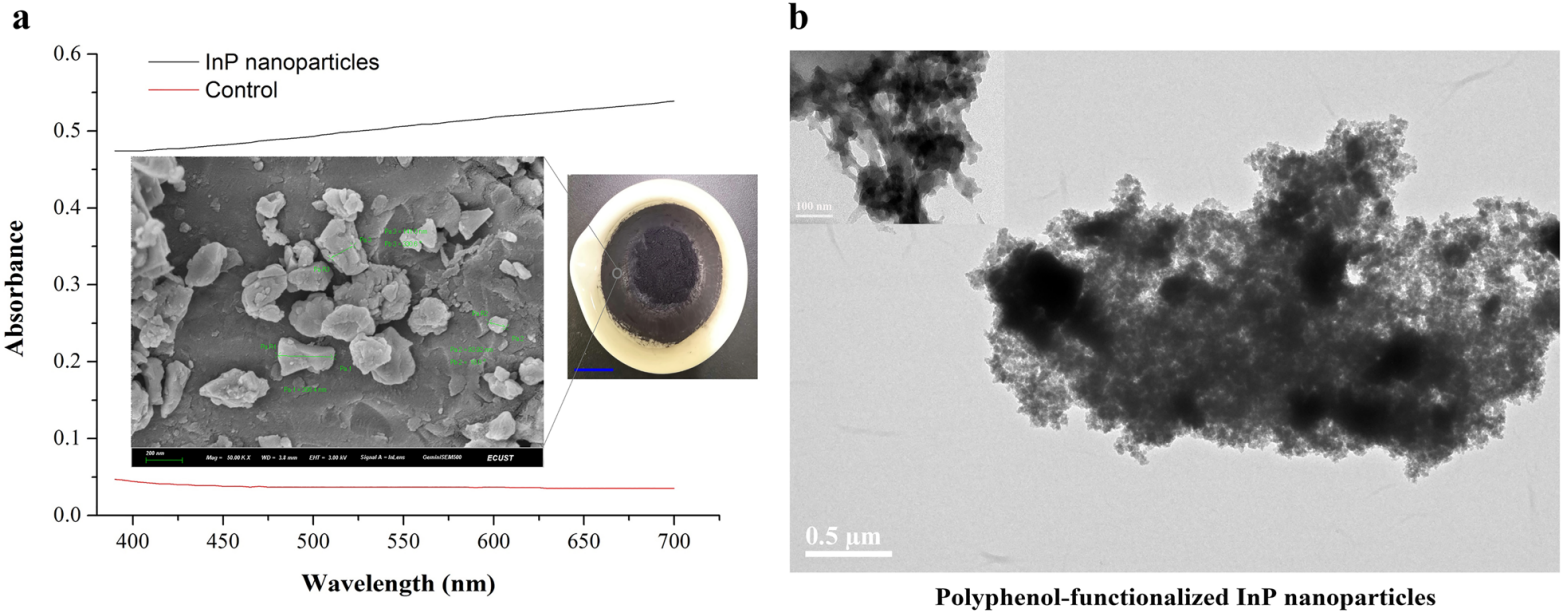
Characterization of InP nanoparticles. **a** UV–Vis spectrum of InP nanoparticles suspension in ddH_2_O, and the control was spectrum of ddH_2_O. SEM image shows the morphology of InP after grinding and its size is distributed in 20–350 nm with less than 500 nm. Blue scale bar is 1 cm. **b** TEM image shows that InP nanoparticles were functionalized via (tannic acid)-based coating. Tannic acid and InP form supramolecular networks on the surface of the nanoparticles resulted in InP is negatively charged

InP nanoparticles between 20 and 500 nm with negative charges are important for the realization of biohybrid systems. Therefore, 3–20 mesh InP powders were manually ground in a mortar for 20–40 min to obtain nanoparticles with diameters less than 500 nm. The ground InP powders were washed three times with ddH_2_O and then dispersed into suspension by ultrasound for 15 min. After centrifugation and attachment to the tube wall, the InP particles were characterized by SEM to determine their size (Fig. 5a). Then, InP nanoparticles and tannic acid solutions were mixed to form an inorganic nanoparticle suspension. Polyphenol-functionalized InP nanoparticles were incubated with 70% ethanol for 10 min and washed with ddH_2_O 3 times. TEM images showed that tannic acid and InP formed supramolecular networks on the surface of the InP nanoparticles (Fig. 5b). This result indicated that the negatively charged tannic acid made the surface of the InP nanoparticles negatively charged. Sonication was applied to disperse the polyphenol-functionalized InP nanoparticles in the suspension to obtain monodispersity of the nanoparticles for uniform adsorption on the cell surface.

**Fig. 6:**
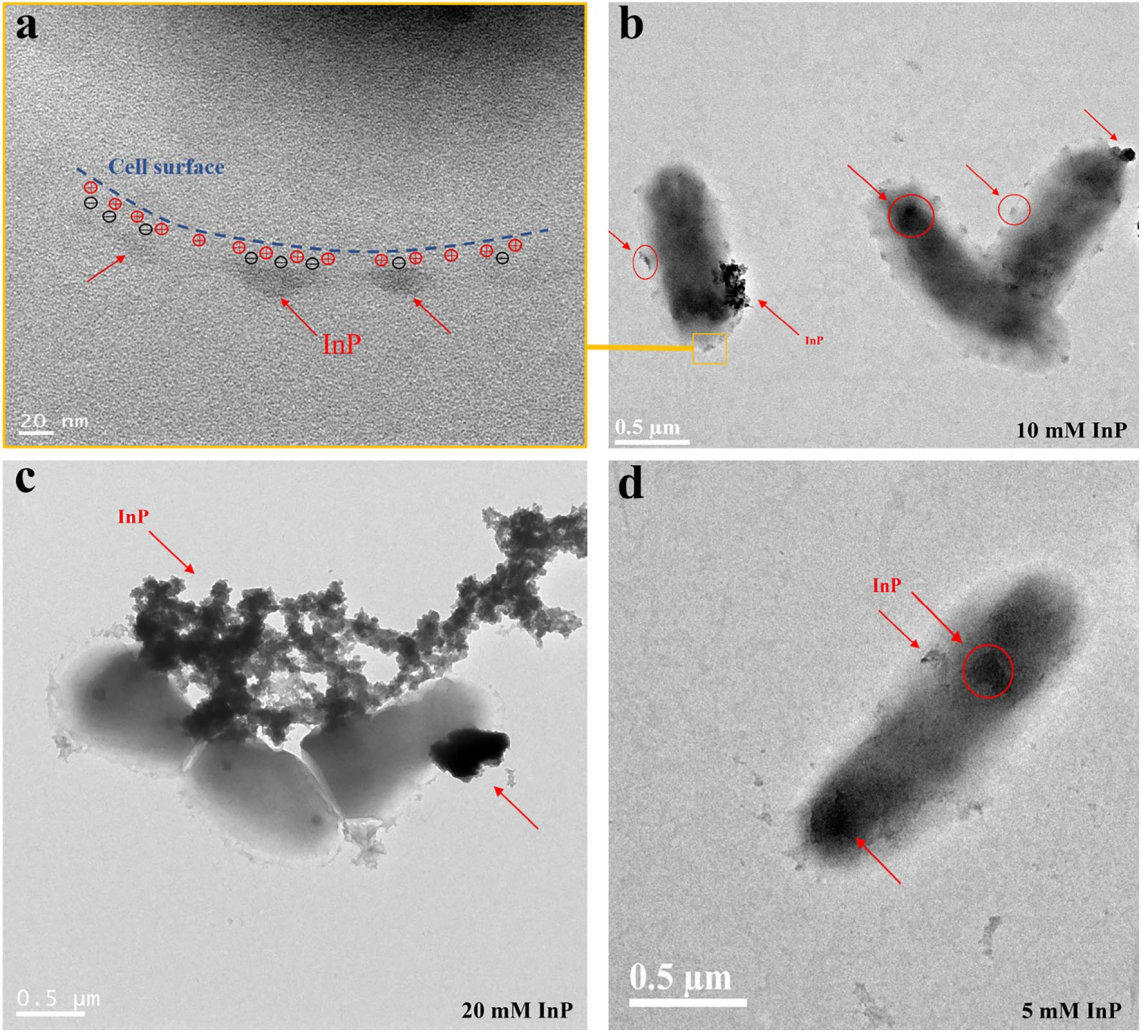
Characterization of *M. neoaurum*-InP biohybrids. TEM images show the overview of biohybrids, and InP of negative charges was adsorbed on the surface of MNR-08 with positive charges. **a** Schematic representation of the biohybrids based on the electrostation. **b**–**d** Show the assembly results of InP and 20 g/L MNR-08 with different contents, respectively

It is essential for the assembly process of biohybrids that a positive charge should enable electrostatic adsorption on the cell surface. Logarithmically metaphase-engineered *M. neoaurum* was harvested and washed with PBS 3 times. Positively charged PAH was mixed with cells so that PAH was used to adsorb on the surface of the cell. The functionalized cells were washed with ddH_2_O three times to remove unabsorbed PAH. The assembly process of biohybrids can occur after mixing polyphenol-functionalized InP nanoparticles with PAH-functionalized cells. TEM images show that InP nanoparticles with negative charges were adsorbed on the surface of cells with positive charges (Fig. 6a). Combined with Additional file 1: Fig. S12, Fig. 6b shows the assembled biohybrids of resting cell fermentation, which were chosen because the cell surface was evenly coated with nanoparticles. Figure 6c suggests that 20 mM InP nanoparticles easily form a barrier equivalent to cell wall thickening and cause difficulties in substrate uptake and electron transfer. However, the surface of cells cannot adsorb sufficient nanoparticles (Fig. 6d), indicating that photoelectric conversion from the surface to the cytoplasm is inefficient.

### Decoupling NADPH regeneration by light-driven electron transport

**Fig. 7:**
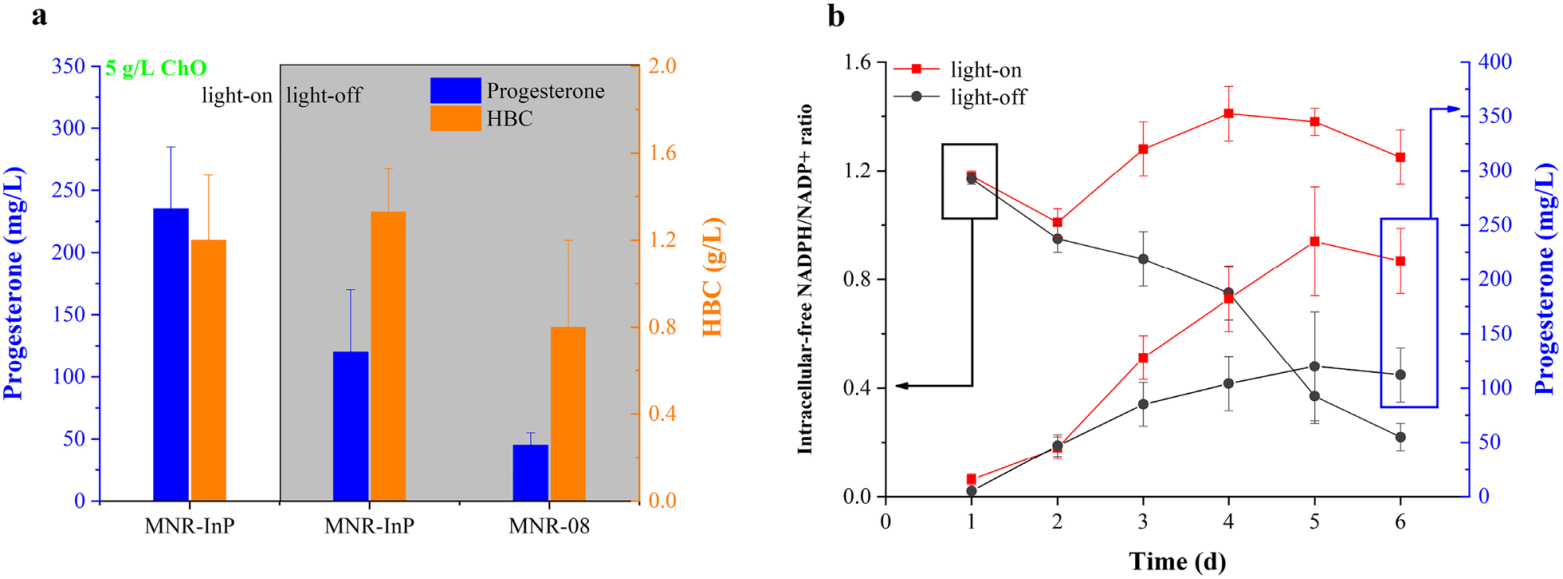
Improved progesterone production by light-driven NADPH regeneration. **a** Comparison of steroid titers in MNR-InP biohybrids and (MNR-08)-only resting conversions for 5 days in the flasks with light-on and light-off conditions. **b** Cytosolic-free NADPH regeneration and progesterone production profiles in light-on and light-off conditions

Figure 2b, c shows that exogenous addition of NADPH can improve the progesterone titer. Additionally, coupling NADPH regeneration was used as a common approach, such as overexpression of glucose-6-phosphate dehydrogenase (G6PDH), which faces byproducts requiring downstream separation and limited regeneration numbers. The intracellular NADPH/NADP^+^ ratio was maintained at a relatively low level by the overexpression of G6PDH, and only 29 mg/L progesterone was produced (Additional file 1: Fig. S13). In contrast, decoupling NADPH generation from other metabolites may enable high production of desired products and lower byproducts (Guo et al. 2018; Wang et al. 2017). A novel method was attempted involving light-driven NADPH regeneration in biohybrids of *M. neoaurum*, where InP light-harvesting semiconductor nanoparticles attached to the surface of bacteria were able to provide reducing equivalents to central metabolic processes (Sakimoto et al. 2015). In other words, electrons flowing from illuminated MNR-surface-bound InP to NADP^+^ inside the bacteria regenerated NADPH (Fig. 1). A high availability of cytosolic NADPH via photon energy conversion (Xu et al. 2019) directly facilitates the production of progesterone, and the progesterone titer produced by this method should be higher than that obtained by overexpressing G6PDH. The effect of light on steroid products was investigated by illuminating resting MNR-InP transforming ChO (Fig. 7a). The results showed that light not only enhanced the titer of progesterone, but also strengthened the titer of the intermediate HBC, which indicated that the illuminated semiconductor can not only transfer electrons for NADP^+^, but also provide electrons for other substances in the cell (Additional file 1: Fig. S14). This conclusion is consistent with the energy-efficient production of the metabolite shikimic acid and its precursor 3-dehydroshikimic acid (Guo et al. 2018).

To determine whether the progesterone increase was due to NADP^+^ reduction, the NADPH/NADP^+^ ratio was tested in cytosolic MNR-InP. Figure 7b shows the highest NADPH/NADP^+^ ratios in the light-on system. These results support that illuminated *M. neoaurum* surface-assembled InP nanoparticles can drive NADPH regeneration in the cytoplasm and enhance steroid production. However, it was difficult to achieve complete darkness in a shaker, which was the cause of the also-increasing steroid yield with lights off. There was another hypothesis for the increased steroid production. PAH functionalization, such as functionalized PHB (poly(3-hydroxybutyrate), is crucial for the high conversion rate by serving as steroid storage, which may mean that the degradation of progesterone is reduced (Gerber et al. 2015). Additionally, the biotransformation trended to a dynamic balance, and the ratio of NADPH/NADP^+^ decreased after 5 days of fermentation, mainly due to the thickening of the cell wall making electron transfer difficult, and the efficiency of substrate uptake and product efflux decreased. Similarly, too much polyphenol-functionalized InP adsorbed on the surface of MNR-08 to form a dense barrier, similar to a thickened cell wall, which hindered electron transfer (Fig. 6c and Additional file 1: Fig. S12). Additional file 1: Fig. S15 suggests that brighter light did not increase progesterone production, but affected the metabolism of steroids and gave rise to cell metabolism disorders. It was also reported that strong light destroyed the cell wall and inhibited the metabolism of the bacteria (Wang et al. 2021). In summary, the illuminated MNR-InP biohybrids drove intracellular NADPH regeneration and facilitate sterol conversion, resulting in a progesterone titer up to 235 ± 50 mg/L, 5.2 and 9.4 times as much as the production titers of MNR-08 and the work previously reported by Donova and Egorova (2012) (Strizhov et al. 2014).

## Conclusions

A green and sustainable approach was established to produce progesterone directly from sterols via the addition of a bovine P450 module in *M. neoaurum*. Concretely, this approach overcame the heterologous interdependency between CYP11A1 and reductase partners, and the module was successfully expressed in *M. neoaurum* to obtain an initial progesterone titer of 2.8 mg/L. To enhance the progesterone titer, the optimization of electron flow and light-driven NADPH regeneration were used together, leading to an 84-fold increase in yield by resting conversion, up to 235 ± 50 mg/L. These conclusions provide a perspective to promote metabolic efficiency for other low-efficiency P450s.

### Supplementary Information

**Additional file 1: Table S1.** Strains and plasmids. **Table S2.** Primers. **Fig. S1.** Localization of CYP11A1 fused GFP were observed by laser scanning confocal microscope. **Fig. S2.** P450 N-terminal modifications, truncations, F-G loop deletions, site-mutations and sequence alignment. **Fig. S3.** Transformation results of HBC, ChO, and PS by MNR-04. **Fig. S4.** Comparison of retention time (RT) of biotransformation products by HPLC (Original images). **Fig. S5–S9.** The results of MS and NMR of progesterone. **Fig. S10.** Antibacterial effect of progesterone for MNR. **Fig. S11.** Metabolic analysis of the effects of progesterone on steroids degradation by MNR fermentation for 5 days. **Fig. S12.** Effects of InP concentrations on progesterone production by MNR-InP biohybrid assembly. **Fig. S13.** Estimation and comparison of intracellular NADPH/NADP^+^ ratio, and corresponding to progesterone titer based on overexpressing gene *g6pdh*. **Fig. S14.** Effect of illuminated resting mnr-InP biohybrids in the phosphate buffer on production HBC. **Fig. S15.** Obtained progesterone in MNR-InP biohybrids under the illumination with different LED power intensities.

## Data Availability

All data generated or analyzed during this study are included in this article (and its additional information files).
