# Supplementary material for: Light-driven progesterone production by InP–(M. neoaurum) biohybrid system
Source: Bioresour Bioprocess. 2022 Sep 1;9(1):93. doi: 10.1186/s40643-022-00575-7 (PMC10992907; doi:10.1186/s40643-022-00575-7)
Supplement: Supplementary file 1 — Additional file 1: Table S1. Strains and plasmids. Table S2. Primers. Fig. S1. Localization of CYP11A1 fused GFP were observed by laser scanning confocal microscope. Fig. S2. P450 N-terminal modifications, truncations, F-G loop deletions, site-mutations and sequence alignment. Fig. S3. Transformation results of HBC, ChO, and PS by MNR-04. Fig. S4. Comparison of retention time (RT) of biotransformation products by HPLC (Original images). Fig. S5–S9. The results of MS and NMR of progesterone. Fig. S10. Antibacterial effect of progesterone for MNR. Fig. S11. Metabolic analysis of the effects of progesterone on steroids degradation by MNR fermentation for 5 days. Fig. S12. Effects of InP concentrations on progesterone production by MNR-InP biohybrid assembly. Fig. S13. Estimation and comparison of intracellular NADPH/NADP+ ratio, and corresponding to progesterone titer based on overexpressing gene g6pdh. Fig. S14. Effect of illuminated resting mnr-InP biohybrids in the phosphate buffer on production HBC. Fig. S15. Obtained progesterone in MNR-InP biohybrids under the illumination with different LED power intensities. [file 40643_2022_575_MOESM1_ESM.docx]

**Additional file 1**

**Light-driven progesterone production by InP-(*M. neoaurum*) biohybrid system**

Kun Liu, Feng-Qing Wang ⃰, Ke Liu, Yunqiu Zhao, Bei Gao, Xinyi Tao, and Dongzhi Wei ⃰

⃰ Corresponding authors,

E-mail: fqwang@ecust.edu.cn (Feng-Qing Wang),

dzhwei@ecust.edu.cn (Dongzhi Wei).

State Key Laboratory of Bioreactor Engineering, Newworld Institute of Biotechnology, East China University of Science and Technology, 130 Meilong Road, Shanghai, 200237, China.

**Table S1**

**Table S1 The key plasmids and strains used in this paper.**

| **Names** | **Descriptions** |
| --- | --- |
| **Plasmids** | - |
| pMV261 | Shuttle vector of *mycobacterium* and *E. coli,* Promoter *hsp60*, *Kan^R^* |
| pMV261-CYP11A1 | pMV261 carrying gene *cyp11a1* under the hsp60 promoter, *Kan^R^* |
| pMV261-CYP11A1-NT | pMV261 carrying gene *cyp11a1*-nt, N-terminal truncated 38 amino acids |
| pMV261-mCYP11A1 | pMV261 carrying gene *mcyp11a1*, N-terminal truncated 38 amino acids, deleted F-G loop, and K193E |
| pMV261-mCYP11A1-ADR-ADX | pMV261 carrying gene *mcyp11a1-adr-adx* via tandem expression |
| pMV261-mCYP11A1-pADR-ADX | pMV261 carrying gene *mcyp11a1-padr-adx* via tandem expression, the *padr* is from porcine |
| pMV261-mCYP11A1-ARH1-ADX | pMV261 carrying gene *mcyp11a1-arh1-adx* via tandem expression, arh1, adrenodoxin reductase-related homolog, is an essential yeast protein in mitochondrial inner membrane |
| pMV261-mCYP11A1-L-ADR-ADX | pMV261 carrying gene *mcyp11a1-l-adr-adx* via tandem expression, L:linker (GGGGSGGGGS) |
| pMV306-ARH1 | pMV306 carrying gene *arh1* under the hsp60 promoter, Hyg^R^ |
| pMV306-G6PDH | pMV306 carrying gene *g6pdh* under the hsp60 promoter, Hyg^R^ |
| pMV261-mCYP11A1-GFP | pMV261 carrying gene *mcyp11a1-gfp*, these two genes were ligated directly into fusion protein |
| **Strains** | - |
| DH5α | *E. coli* cloning host |
| *M. neoaurum* ATCC 25795 | *M. neoaurum* type strain |
| MNR | *KshA1, hsd4A, FadA5，kstD1，kstD2* and *kstD3* deleted in *M. neoaurum* ATCC 25795 |
| MNR-01 | MNR with plasmid pMV261 |
| MNR-02 | MNR with plasmid pMV261-CYP11A1 |
| MNR-03 | MNR with plasmid pMV261-mCYP11A1 |
| MNR-04 | MNR with plasmid pMV261-mCYP11A1-ADR-ADX |

**Continued Table S1**

| MNR-05 | MNR with plasmid pMV261-mCYP11A1-pADR-ADX |
| --- | --- |
| MNR-06 | MNR with plasmid pMV261-mCYP11A1-ARH1-ADX |
| MNR-07 | MNR with plasmid pMV261-mCYP11A1-L-ADR-ADX |
| MNR-08 | MNR with plasmid pMV261-mCYP11A1-L-ADR-ADX, and plasmid pMV306-ARH1 (The expression cassette of the ARH1 gene was integrated into the corresponding *attB* sites of MNR genome.) |
| MNR-09 | MNR with plasmid pMV261-CYP11A1-GFP |
| MNR-10 | MNR with plasmid pMV261-mCYP11A1-GFP |
| MNR-11 | MNR with plasmid pMV261-mCYP11A1-L-ADR-ADX, and plasmid pMV306-G6PDH (The expression cassette of the G6PDH gene was integrated into the corresponding *attB* sites of MNR genome.) |
| MNR-InP | MNR-08 and InP nanoparticles were assembled into biohybrids |
| mnr-InP | MNR and InP nanoparticles were assembled into biohybrids |

**Tips:** Fonts underlined indicate a fusion protein.

**Table S2**

**Table S2 Main oligonucleotides used in this study.**

| **Name（Primers）** | **DNA sequence （sequence 5` - 3`）** |
| --- | --- |
| CYP11A1-UP | GGAATCACTTCGCAATGGCCAAGATGCTGGCCCGCGGCCTGCCG |
| CYP11A1-DOWN | ACGCTAGTTAACTACGTCGACGGCCTGCGGCGGGTCCTGGTT |
| Sequencing-UP | GGTGGTTGTGGTGATGTACGTG |
| Sequencing-DOWN | CTTGTGCAATGTAACATCAGAG |
| CYP11A1-NT-UP | AGGAATCACTTCGCAATGGCCAACTCGACCAAGACCCCGCG |
| CYP11A1-NT-DOWN | ACGCTAGTTAACTACGTCGACGGCCTGCGGCGGGTC |
| Infusion1-F | CCGCCGCAGGCCTGAGGAGGAAATGGCCCCGCGCTGCTG |
| Infusion1-R | GGGCCATTTCCTCCTCAGGCCTGCGGCGGGTCCTGGTTG |
| F-G loop-UP | GCGACATCAAGGAGATGCTGGAGGAGACCGTG |
| 193E-DOWN | TCTCCTCCAGCATCTCCTTGATGTCGCCCACGAACTCG |
| F-ADX-F | ATGCTGGAGGAGACCGTGAACCCGG |
| Infusion2-R | GGGCCATTTCCTCCTCAGGCCTGCGGCGGGTCCTGGTTG |
| Infusion2-F | CCGCCGCAGGCCTGAGGAGGAAATGGCCCCGCGCTGCTG |
| Infusion3-F | ACCCGCCGCAGGCCTGAGGAGGAAATGTCGTTCGTGCAG |
| Infusion3-R | ACGAACGACATTTCCTCCTCAGGCCTGCGGCGGGTCCTGG |
| Infusion4-F | GGAGGGCATCTGAGGAGGAAATGGCCGCGCGCCT |
| Infusion4-R | GCCATTTCCTCCTCAGATGCCCTCCACGCCGGACCACA |
| ADX-DOWN | GCTAGTTAACTACGTCGACTCACTCGATCTTCGACGAGTTCATG |
| linker-ADR-UP | CCGCCGCAGGCCGGCGGCGGCGGCTCGGGCGGCGGCGGCTCGATGGCCCCGCGCTGCTG |
| linker-down | GAGCCGCCGCCGCCGGCCTGCGGCGGGTCCTGGTTGAACG |
| G6PDH-F | ATGAGCACAGCCGAGGCATC |
| G6PDH-R | TCACGGTCGCCGCCACTC |

**Tips: The key restriction enzyme recognition site is underlined.**

**Figure S1**

**
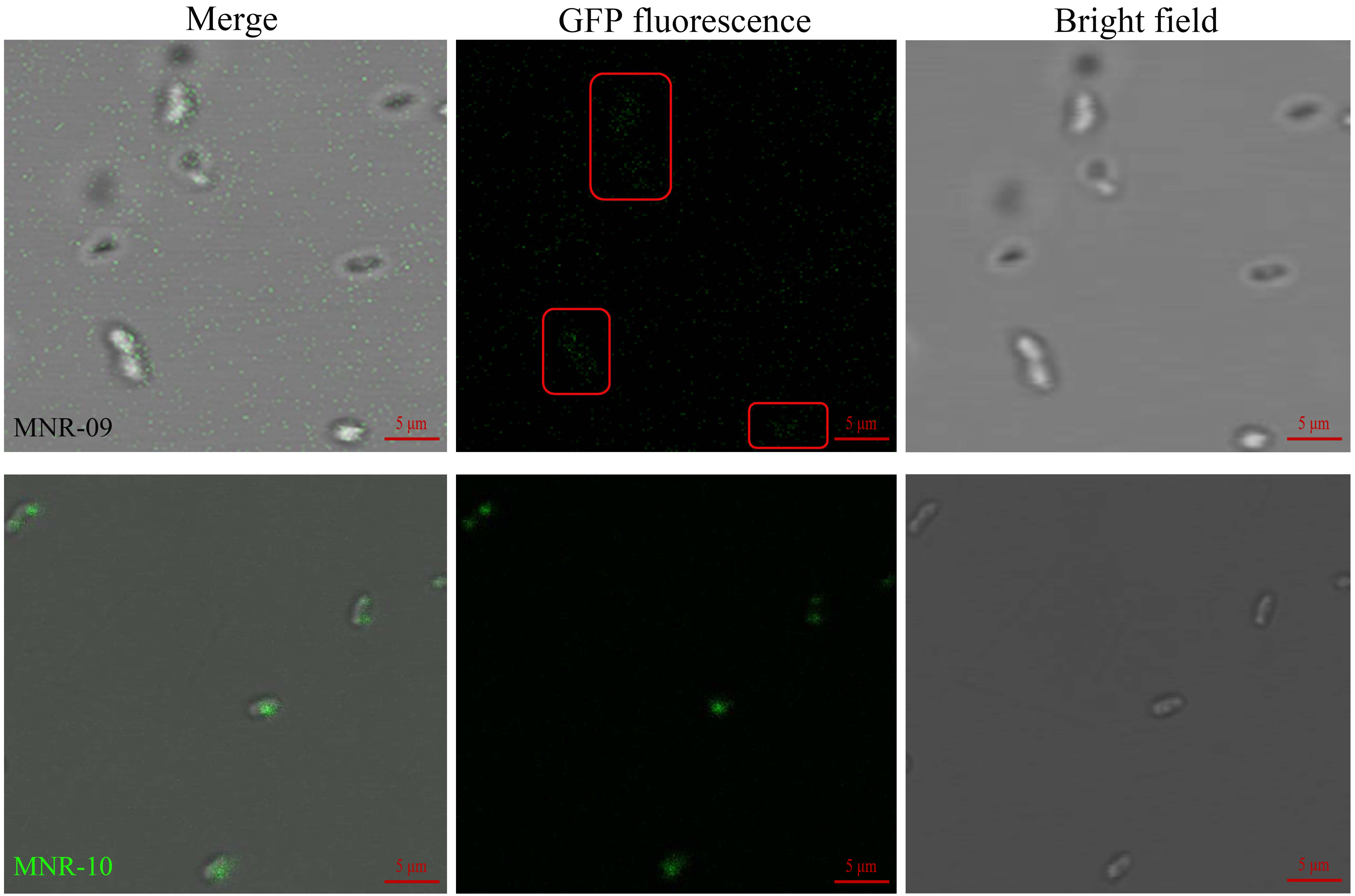
**

**Fig. S1** **Localization of CYP11A1 fused GFP were observed by laser scanning confocal microscope (LEICA TCS SP8, x60 water immersion objective).** CYP11A1-GFP and mCYP11A1-GFP were expressed in MNR-09 and MNR-10, respectively.

**Figure S2**

**Fig. S2** **P450 N-terminal modifications, truncations, F-G loop deletions, site-mutations and sequence alignment.**

**Figure S3**

**
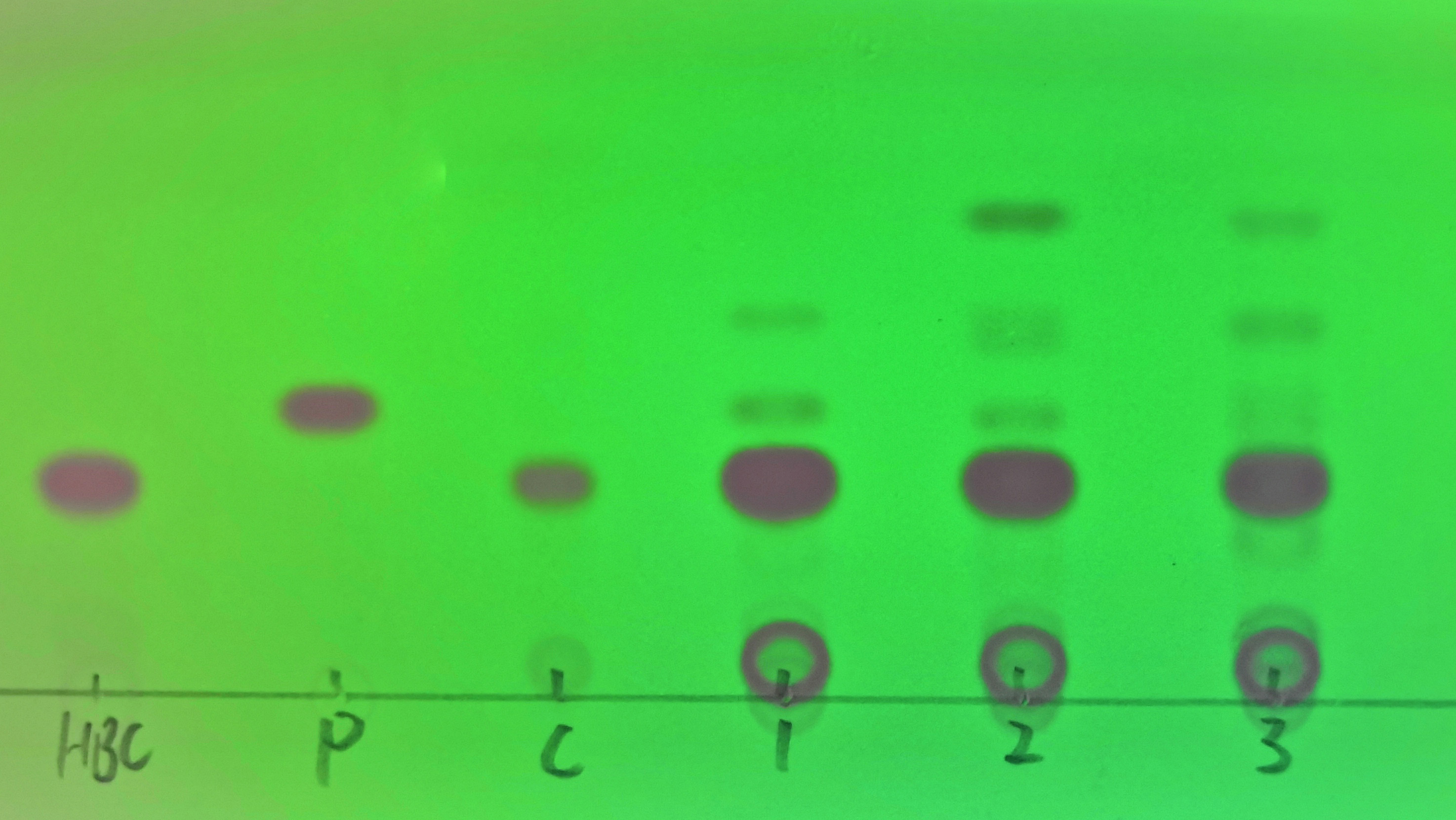
**

**Fig. S3 Transformation results of HBC, ChO, and PS by MNR-04.** The TLC spectrogram was showed under 254 nm. Lane HBC: standard HBC; Lane P: standard progesterone; Lane C (control group): the conversion result of PS (1 g/L) to HBC by the strain MNR-01. Lanes 1-3: The substrates were HBC, ChO, and PS, respectively. It needs to be mentioned that PS shows no color under 254 nm. The Rf value of progesterone was 0.43±0.05.

**Figure S4**

**

**

**Fig. S4** **Comparison of retention time (RT) of biotransformation products by HPLC (Original images).** The blank (MNR-01) and control (MNR-01) were added 0 g/L and 1 g/L PS into medium, respectively. 1 g/L HBC was added into medium as conversion substrate of the recombination strain (MNR-04).

**Figure S5**

**
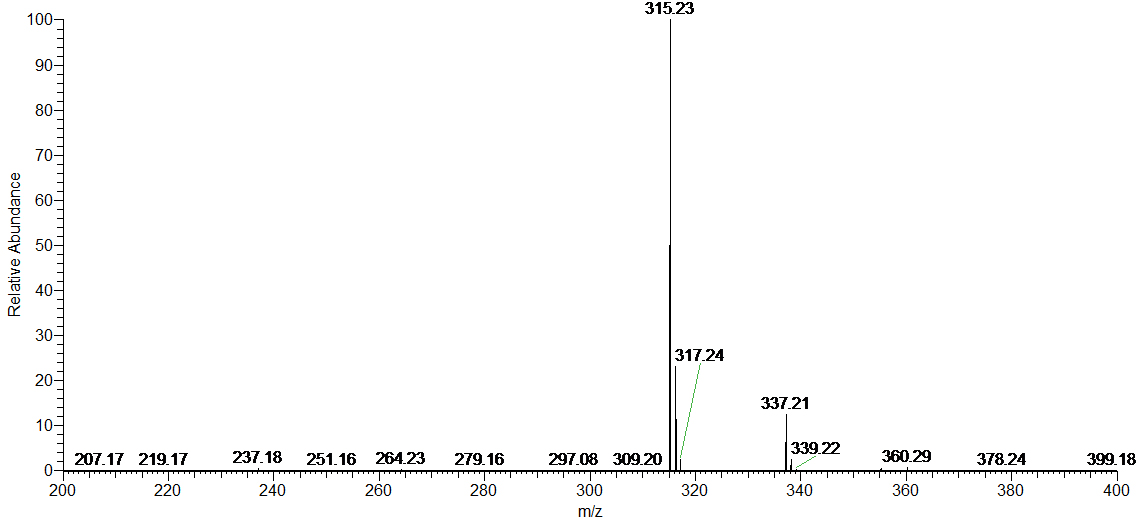
**

**Fig. S5** **UHPLC-MS for RT (retention time) 6.27 min.** This was the standard progesterone (m/z=315.23 (M+H^+^).

**Figure S6**

**
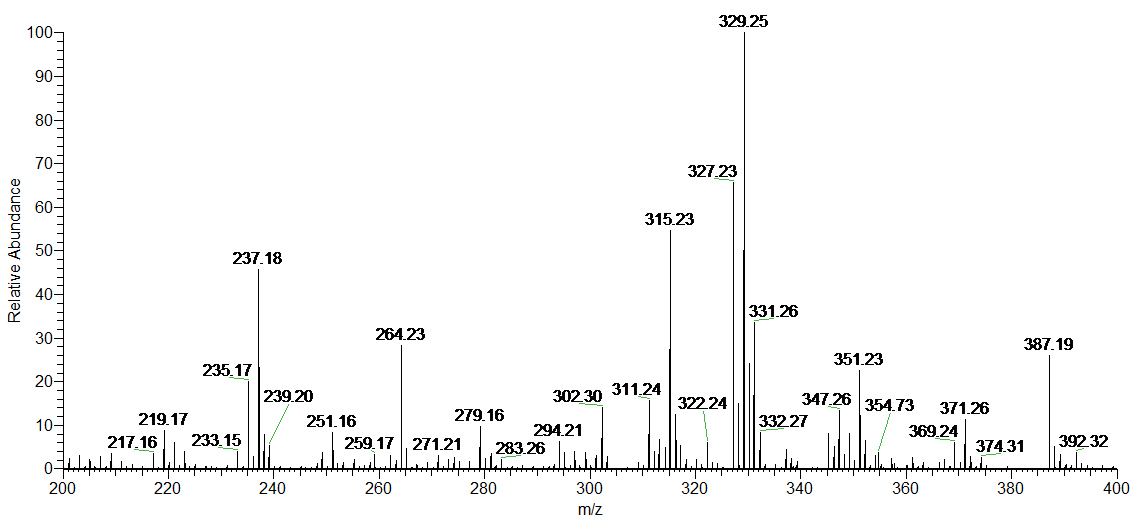
**

**Fig. S6 UHPLC-MS for RT 6.27 min.** This was the obtained progesterone by MNR-04 conversion HBC for 5 days [progesterone: (m/z=315.23 (M+H^+^), HBC: (m/z=331.26 (M+H^+^)].

**Figure S7**

**
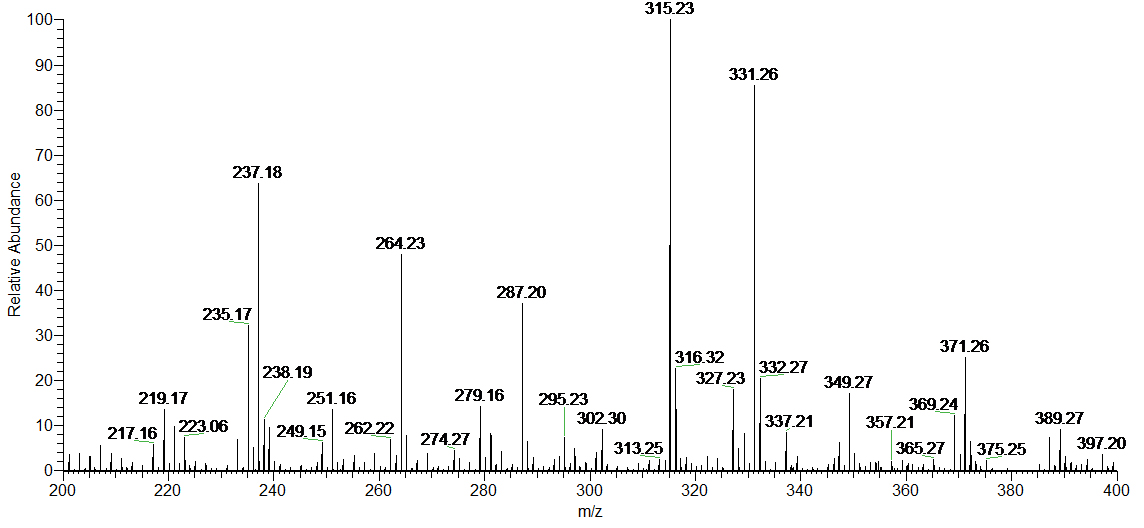
**

**Fig. S7 UHPLC-MS for RT 6.27 min.** This was the obtained progesterone by MNR-04 conversion cholesterol for 7 days [progesterone: (m/z=315.23 (M+H^+^), HBC: (m/z=331.26 (M+H^+^)].

**Figure S8**


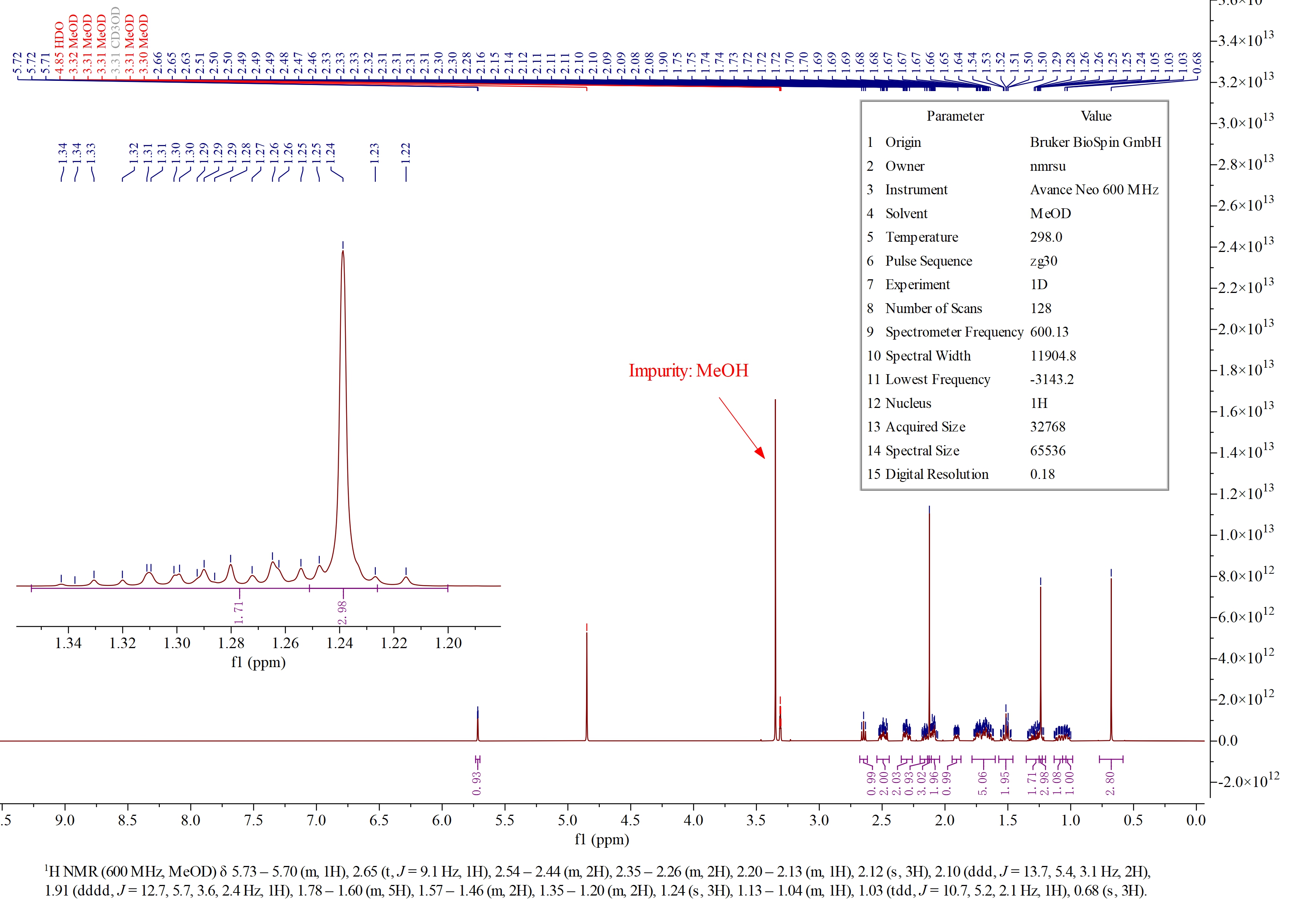


**Fig. S8** **^1^H-NMR (600 MHz, Methanol-*d_4_*) spectrum of purified progesterone.**

**Figure S9**


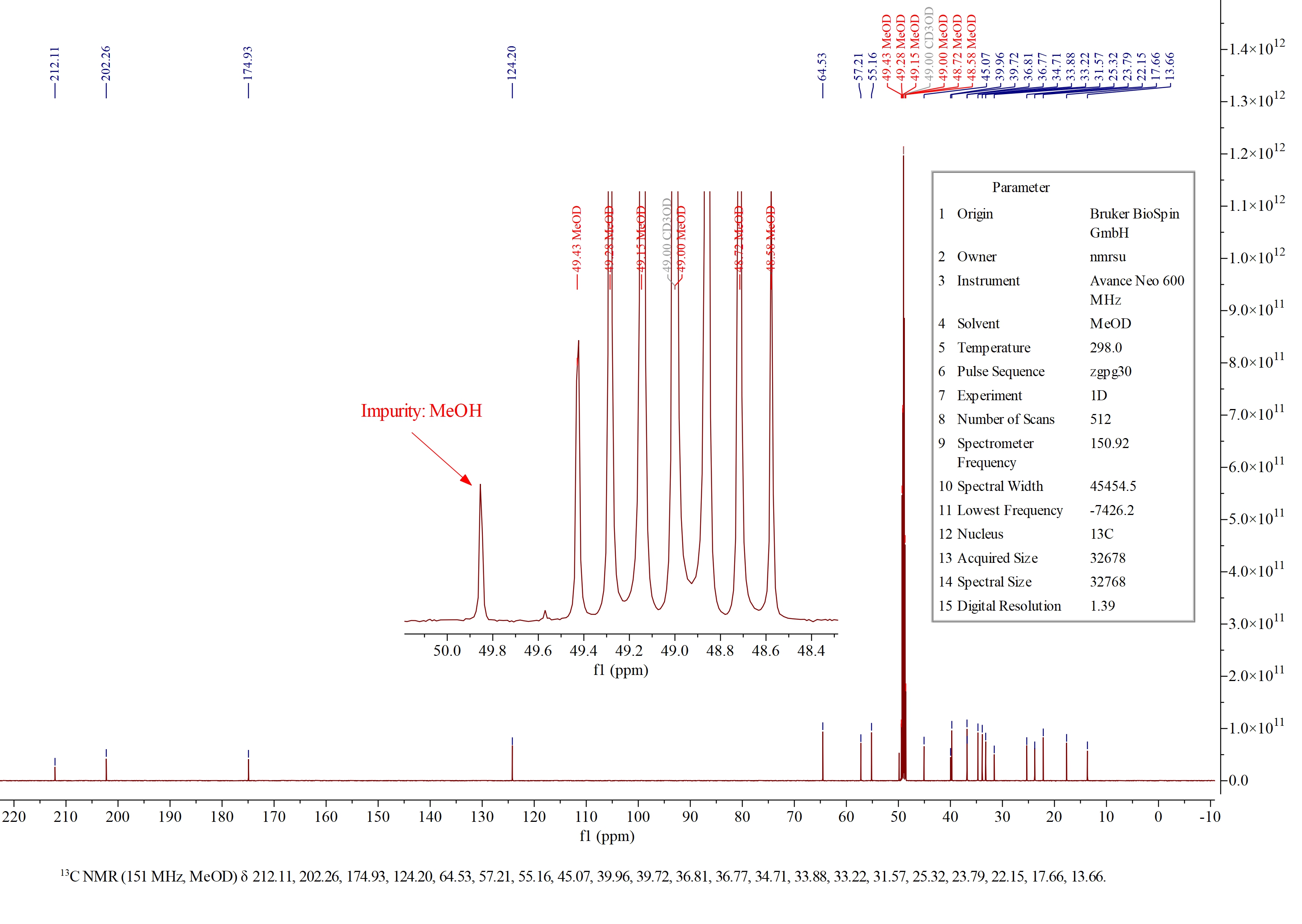


**Fig. S9** **^13^C-NMR (151 MHz, Methanol-*d_4_*) spectrum of purified progesterone.**

**Figure S10**

**
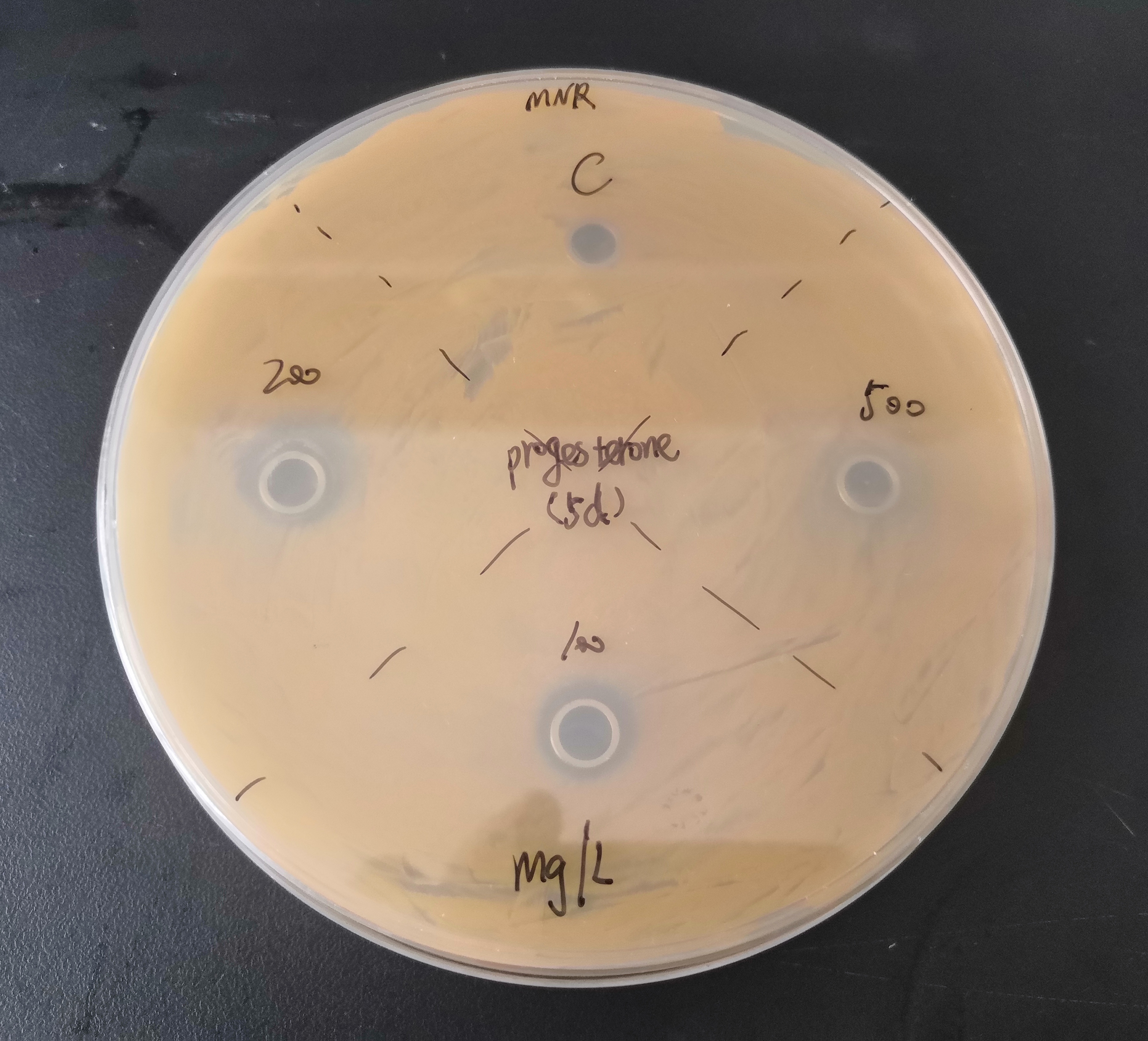

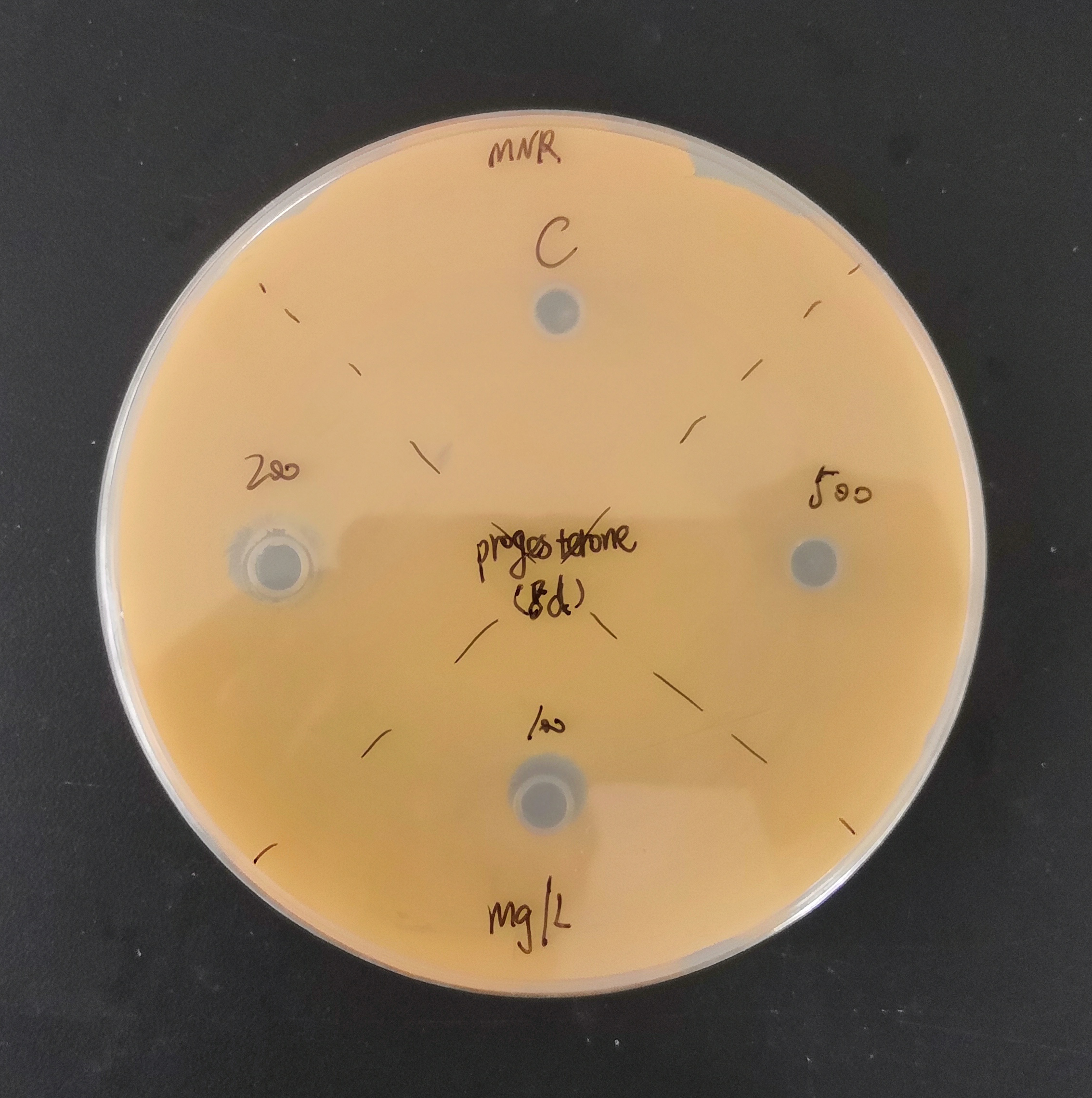
**

**Fig. S10 Antibacterial effect of progesterone for MNR (Original images).** The image**s** show that the 5d inhibition zones (C: control, 8 mm, 100: >10 mm, 200: >10 mm, 500 mg/L: 8 mm) were more than 8d, these results indicated the progesterone was metabolized by MNR, exception for group 500 mainly because progesterone cannot be completely dissolved in the solvent, and maybe its precipitation at the bottom of the oxford cup (Ф8*6*10 mm) hindered the diffusion of the dissolved progesterone.

**Figure S11**


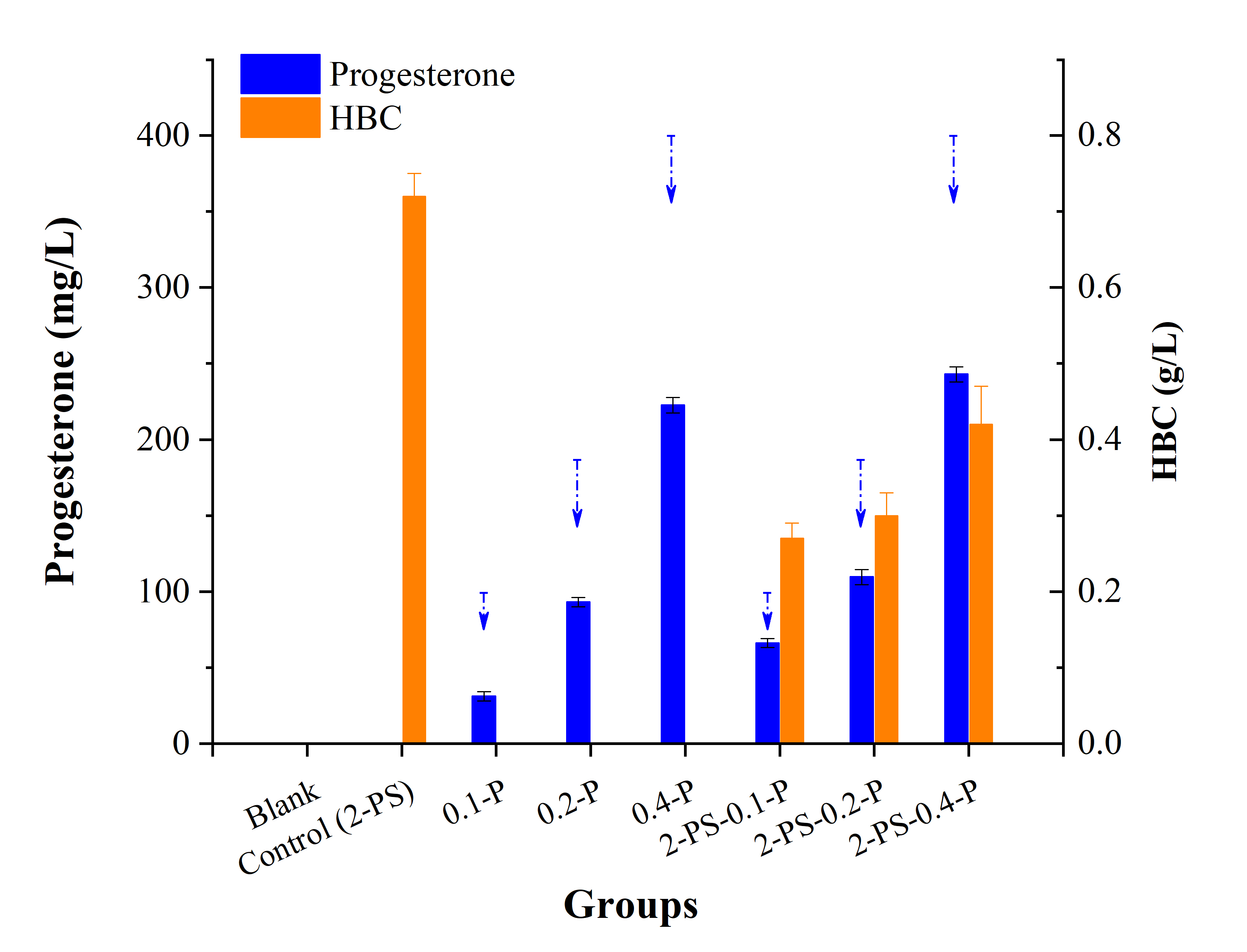


**Fig. S11 Metabolic analysis of the effects of progesterone on steroids degradation by MNR fermentation for 5 days.** The results suggested the MNR had metabolic pathways to degrade progesterone, and the progesterone severely inhibited the metabolic pathways from PS to HBC. The blank means only had the MNR in fermentation medium (MNR02); the control was that the addition of 2 g/L PS into MNR02 by MNR biotransformation. Homoplastically, group 0.1-P and 2-PS-0.1-P were the addition of 0.1 g/L progesterone and 2 g/L PS with 0.1 g/L progesterone into MNR02 by MNR biotransformation, respectively. The blue arrow points decrement of progesterone.

**Figure S****12**

**
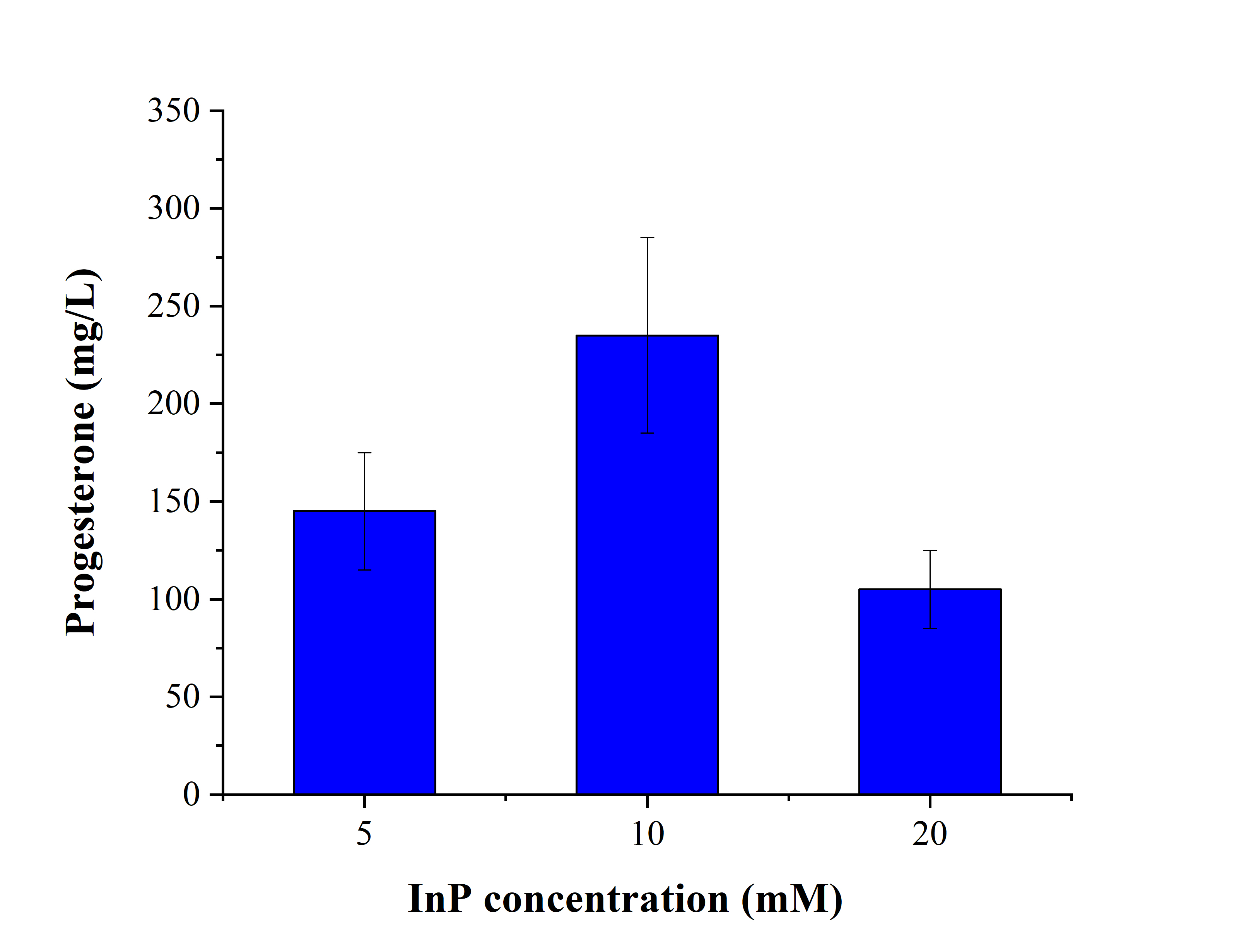
**

**Fig. S12 Effects of InP concentrations on progesterone production by MNR-InP biohybrid assembly.** The assembly of nanoparticles on cell surface can occur after mixing because of electrostatic adsorption. The concentrations of nanoparticles and 20 g/L MNR-08 were assembled after mixing in flasks. Combined with Fig. 6c, the concentration of InP nanoparticles was 20 mM, 10 mM InP nanoparticles and 20 g/L MNR-08 assembling MNR-InP biohybrids were chosen as further studies. Tips: The absorbance of a suspension at 570 nm was used to detect the content of indium phosphide.

**Figure S13**


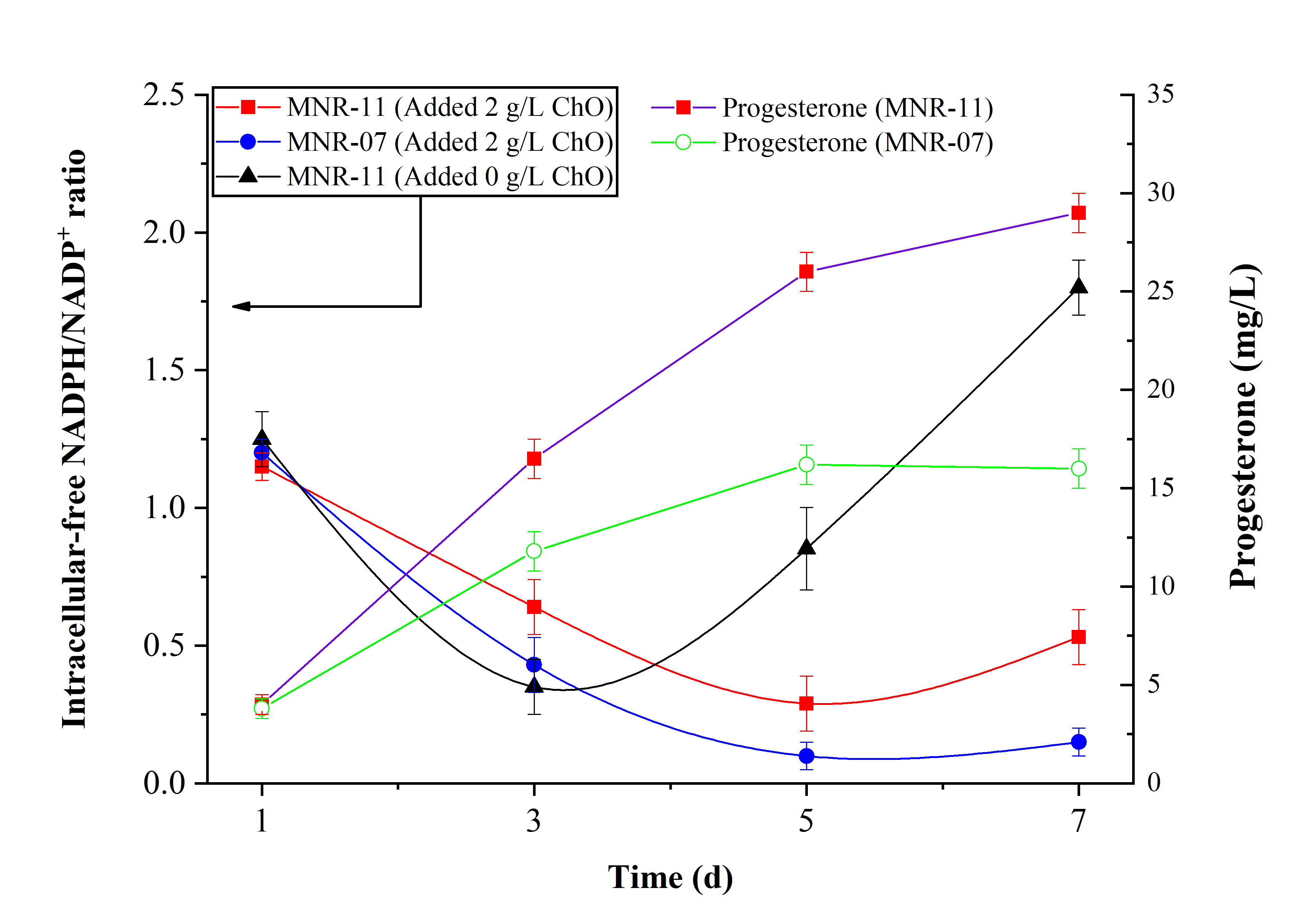


**Fig. S13 Estimation and comparison of intracellular NADPH/NADP^+^ ratio, and corresponding to progesterone titer based on overexpressing gene *g6pdh*.** In order to reduce the burden of plasmid pMV261, and we have only one expression plasmid pMV261 to use, strain MNR-07 was selected as the control strain and host in this enzymatic regeneration of NADPH. Therefore, the expression cassette of the G6PDH was integrated into the *attB* sites of genome by plasmid pMV306.

**Figure S14**

**
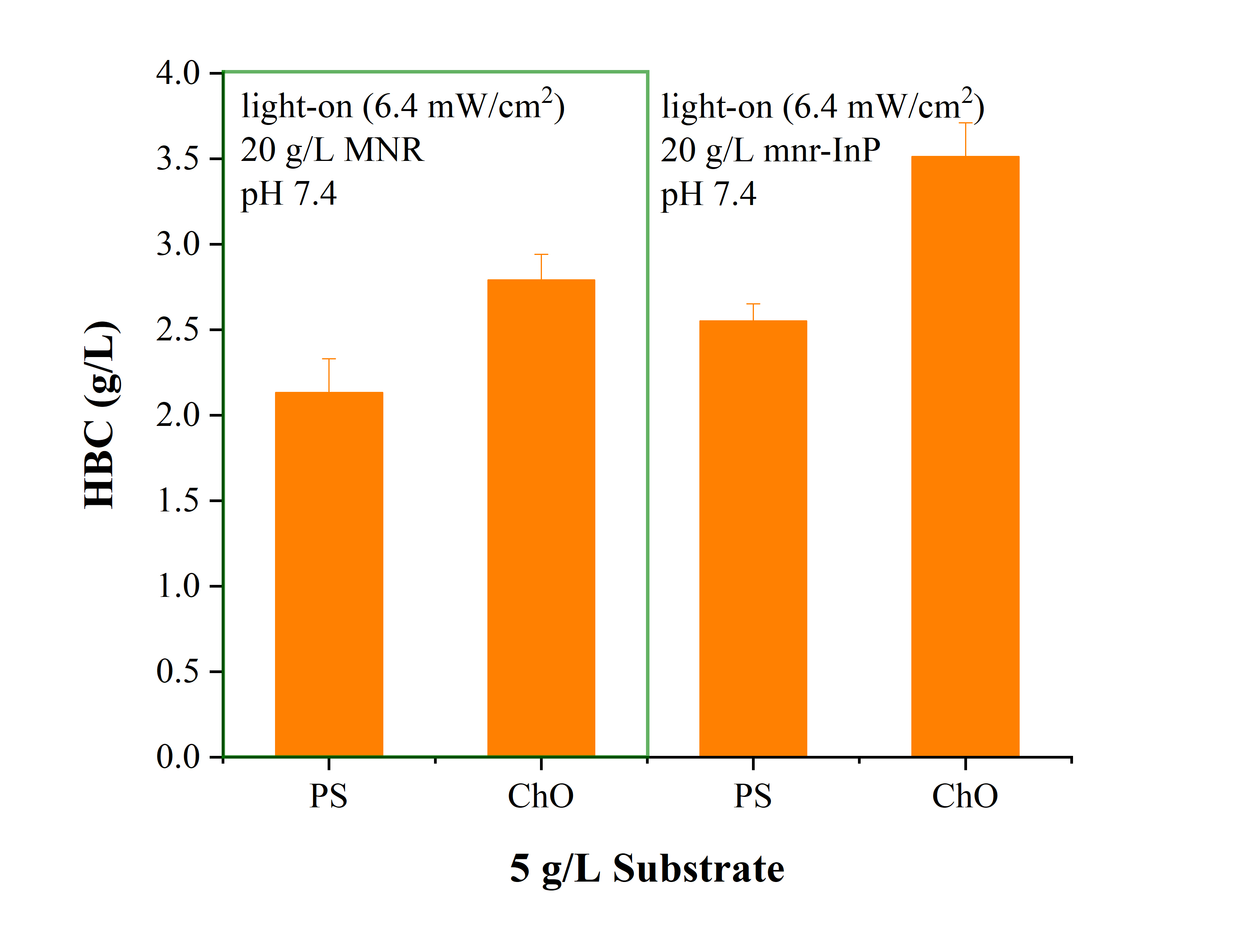
**

**Fig. S14 Effect of illuminated resting mnr-InP biohybrids in the phosphate buffer on production HBC.** The mnr was strain MNR. The result showed that regardless of PS or ChO, the light on mnr-InP enhanced the titer of HBC, which means illuminated InP semiconductor can transfer electrons for metabolic pathway from sterols to HBC to facilitate HBC production.

**Figure S15**

**
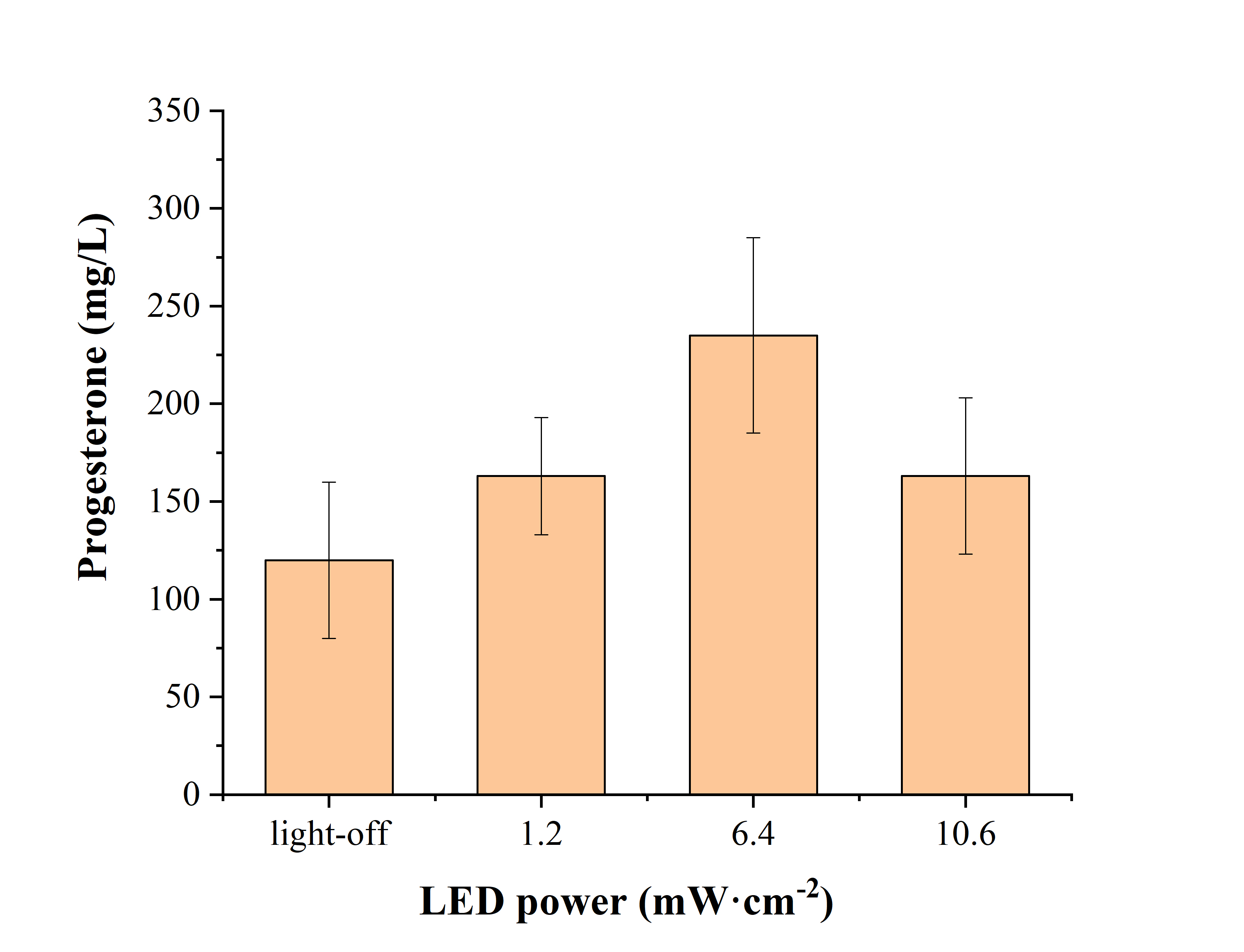
**

**Fig. S15 Obtained progesterone in MNR-InP biohybrids under the illumination with different LED power intensities (Digital Lux Meter AS823).**

Based on reported, we selected 400-760 nm as visible light wavelength, hence we identified that,

1 lux =0.01768 W·m^-2^,

Where lux (lx) is illuminance and the unit of radiant flux density is W·m^-2^.
